# Risks to Recreational Water Users from *Microcystis* spp. and Microcystins in Portishead Lake (SW England) as Determined by LC-MS/MS and Citizen Science Monitoring

**DOI:** 10.3390/toxins18100442

**Published:** 2026-10-13

**Authors:** Andrew D. Turner, Karl J. Dean, Monika Dhanji-Rapkova, Alison O’Neill, Benjamin H. Maskrey, Patrycja Stubbs, David M. Hartnell, Shamina Page, Naomi Baker, Nadine Morrell, John M. Turner, Joan Turner, Florence R. I. Turner, Stephen D. Gregory, Adam M. Lewis, Katy Lewis

**Affiliations:** 1 Centre for Environment, Fisheries and Aquaculture Science (Cefas), Barrack Road, The Nothe, Weymouth DT4 8UB, Dorset, UK; karl.dean@cefas.gov.uk (K.J.D.); monika.dhanji@cefas.gov.uk (M.D.-R.); ben.maskrey@cefas.gov.uk (B.H.M.); patrycja.stubbs@cefas.gov.uk (P.S.); david.hartnell@cefas.gov.uk (D.M.H.); shamina.page@cefas.gov.uk (S.P.); naomi.baker@cefas.gov.uk (N.B.); nadine.morrell@cefas.gov.uk (N.M.); stephen.gregory@cefas.gov.uk (S.D.G.); adam.lewis@cefas.gov.uk (A.M.L.); 2 Portishead Sampling Team, Sage Close, Portishead BS20 8ET, North Somerset, UK; 3 School of Applied Sciences, University of West of England, Frenchay Campus, Coldharbour Lane, Bristol BS16 1QY, UK; 4 Centre for Conservation Ecology and Environmental Sciences, Faculty of Science and Technology, Bournemouth University, Talbot Campus, Poole BH12 5BB, Dorset, UK; 5 Environment Agency, Horizon House, Deanery Rd, Bristol BS1 5AH, UK; katy.lewis@environment-agency.gov.uk

**Keywords:** cyanobacteria, cyanotoxins, lake, microcystins, human health, recreational exposure

## Abstract

Cyanobacteria are microorganisms that can produce natural toxins with significant impacts on human, animal and ecosystem health. Portishead Marine Lake is a small freshwater lake in southwest England frequented by thousands of people each year for recreational activities but was associated periodically with prolific cyanobacterial blooms with potential risk to visitor health. Water samples were tested weekly between January and December 2016 for cyanobacteria and cyanotoxins. Citizen scientists undertook testing for nutrients, dissolved oxygen, pH, temperature and meteorological parameters. Four major blooms of *Microcystis* spp. were observed in the warmer months between May and September and were associated with high concentrations of microcystins. Complex toxin congener profiles were dominated by MC-LR and MC-RR, with lower contributions from MC-WR and MC-YR. Cyanobacterial cell densities and summed microcystin concentrations ranged from 1600 to 5.6 × 10^6^ cells/mL and 0.1 to 18,069 µg/L, respectively, and correlated significantly. Toxin concentrations were positively correlated with nitrate levels and inversely correlated with pH, although mean pH levels did not fall below 7.0. There was little statistical evidence for influence of weather conditions overall, although significant intra-lake differences in cell densities and toxin concentrations were noted. Intervention measures employed by local authorities included the use of barley straw, hydrological manipulation, debris removal and ultrasound treatment. Whilst no statistical assessment of intervention effectiveness was possible without effective control samples, there was some limited evidence for the temporary reduction in toxin levels after hydrological intervention, but none following installation of ultrasound. Overall, the study utilized LC-MS/MS and microscopy to quantify microcystins and *Microcystis* spp., respectively, and evidenced risks to lake visitors and the importance of toxin testing in recreational waters and the use of appropriate risk mitigation approaches. Further work incorporating appropriate experimental controls would be needed to determine the effectiveness of hazard mitigation measures for reducing or eliminating human health threats.

## 1. Introduction

Cyanobacteria are photosynthetic bacteria with near global distribution that can have significant impacts on human, animal and ecosystem health [1,2,3,4]. Some cyanobacteria produce toxins, with a wide range of adverse effects and targets including hepato-, neuro-, cyto-, dermato- and genotoxicity as well as carcinogenicity [5,6,7,8,9]. These can seriously affect human health through their impacts on drinking water quality, uptake of toxins into aquatic foodstuffs and recreational impacts due to risks from body contact, or swallowing or inhaling toxins in the surface waters [10,11,12,13,14].

**Figure 1:**
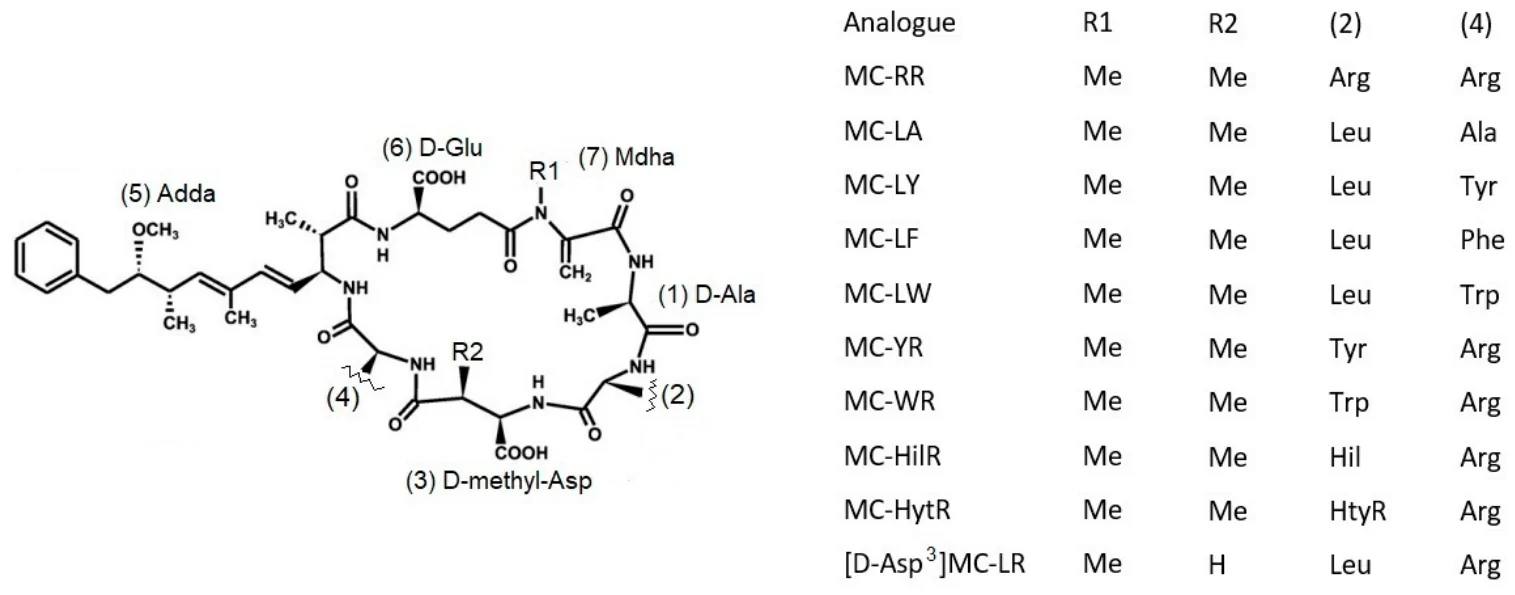
Generalized structure of MCs showing molecular substituents in analogues utilized in this study. Leu—Leucine, Arg—Arginine, Ala—Alanine, Tyr—Tyrosine, Hil—Homoisoleucine, Trp—Tryptophan, Phe—Phenylalanine, Me—Methyl, HtyR—Homotyrosine.

The most commonly encountered and widely studied toxin groups are the microcystins (MCs) (Figure 1), compounds produced mainly by cyanobacteria of the genera *Microcystis*, *Planktothrix*, *Anabaena*/*Dolichospermum* and *Oscillatoria* [15], with toxins linked primarily to hepatotoxicity as well as cardio-, immuno- and developmental toxicity [1,9,16,17].

Given the severity to human health from cyanobacteria, there is a strong need to manage the risks associated with harmful blooms with a coordinated management system [18,19]. Risk management typically requires effective monitoring of water bodies for the assessment of cyanobacterial presence, speciation, cell density and ideally toxin content. Enumeration of potentially toxigenic cyanobacterial species does not, however, guarantee the presence of toxins, with a recent screen of freshwater bodies in England revealing that more than 50% of cyanobacteria samples contained quantifiable levels of toxins [20] with just 13% of these exceeding the WHO medium health threshold of 24 µg/L [21]. Consequently, there is a clear need for quantitation of cyanotoxins in cyanobacterial blooms to determine the true risk to human health.

MC-producing blooms have been well recognized in the UK, most notably following incidents of animal poisonings [11,22,23,24,25,26,27,28], although some human cases have also been published [11,22]. In the United Kingdom (UK), previous studies have highlighted the prevalence of MCs in a high proportion of cyanobacterial blooms [20,23,27], which, together with both increased reports of blooms and the growing popularity of freshwater recreation, results in strong evidence for continued risk to human health [27,29,30,31,32]. More recently, MCs were determined in blooms of *Microcystis*, *Planktothrix*, *Anabaena/Dolichospermum*, *Aphanizomenan* and *Oscillatoria* in freshwater samples from England [20], with concentrations reaching a maximum of 42.7 mg/L.

Portishead Marine Lake is a freshwater water body located in North Somerset, England. The lake is used for recreational boating activities, but no swimming takes place given the shallow water. In recent decades, there have been regular reports of “toxic blue/green algae” in the lake, as well as occasional reports of both dead fish and dead birds [33,34,35]. During 2014 and 2015, periodic blooms were assessed by the Environment Agency (EA) with light microscopy, confirming cyanobacterial species were present, most notably *Microcystis* spp. Some intervention was conducted through the placement of multiple barley straw bales into the water, a measure deemed previously to have beneficial impacts on cyanobacterial blooms [36,37]. As blooms remained periodically through both years, at times access to the lake had to be closed, creating a significant impact on users of the grounds. The lake has been used for many years for boating activities, noting the recognized increases in health effects from boat users on lakes with high cyanobacteria concentrations [38]. The lake also includes a shallow end for young children to paddle in, so there may be higher risks for children playing in near-shore waters where cyanobacterial scums can accumulate [1].

Given the frequency of the blooms, the closures of the Portishead Marine Lake and the concern of locals and visitors, at the beginning of 2016, a study was designed to systematically assess the presence of cyanobacteria within the lake over the course of the year. Whilst light microscopy had been used previously to confirm the species and to enumerate the cell densities, no work had previously been conducted to assess the presence of toxins in the cyanobacterial cells and water. Consequently, this study sought to sample throughout the year from multiple locations around the lake, assessing cyanobacteria presence and analyzing water and algal cells for microcystins. In addition, water chemistry and meteorological measurements were taken using local citizen scientists, with the aim of building a dataset to help determine the potential causative factors for any toxin-producing blooms within the lake and subsequent anticipated impacts on human health.

## 2. Results

### 2.1. Sampling, Data Collection and Environmental Parameters

**Figure 2:**
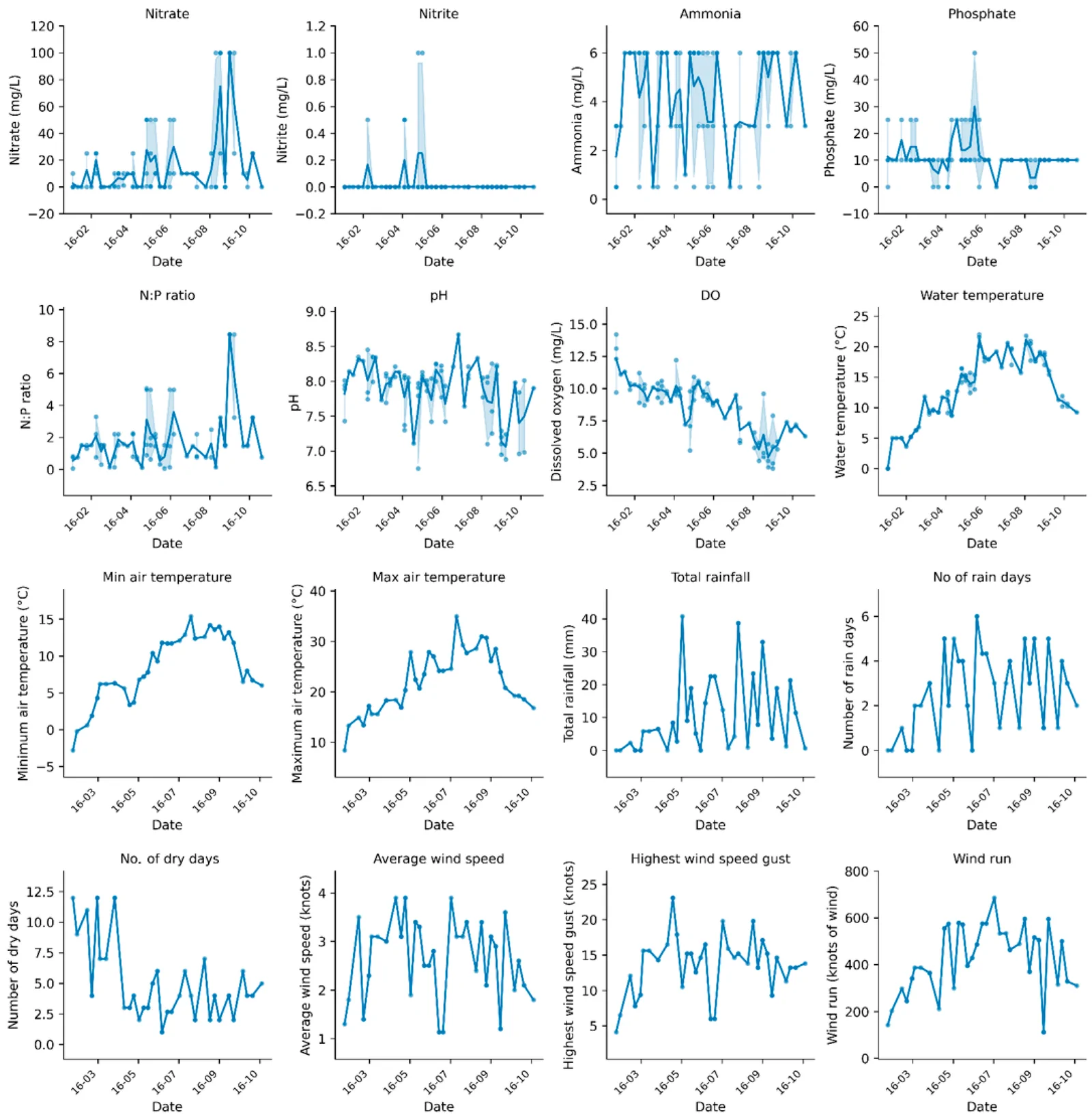
Time series line charts illustrating metrological and environmental parameters. Environmental parameters are shown as daily means with shading representing the 95% percentage interval across sites. Nutrient concentrations measured in mg/L; DO (dissolved oxygen) measured in mg/L; water temperature measured in °C; min air temperature measured in °C; max air temperature measured in °C; total rainfall measured in mm; average wind speed measured in knots; highest wind speed gust measured in knots; wind run measured in knots of wind.

The first samples were taken on 21 January 2016 when the air temperature was 0.5 °C and a significant proportion of the lake contained surface ice. After this point, the air temperature increased slowly through to Spring and Summer, with no further ice observed in the water until 1 December 2016. Figure 2 illustrates the metereological data recorded by citizen scientists throughout the study period. For each week, mean, absolute minimum and maximum, mean daily minimum and mean daily maximum temperatures were found to vary through the four seasons as expected. Mean temperatures ranged from 2.5 °C to 20 °C, with the minimum temperature dropping to −2.8 °C in January and the highest being 35 °C during July. Negative Celsius temperatures and air frosts were only recorded over a total of three recording periods, between 23 January 2016 and 13 February 2016 and between 24 November 2016 and 1 December 2016. Rainfall varied considerably, reaching a maximum of 138 mm rain during a seven-day period at the end of November. Other months with notable amounts of rain were May, June and July. No clear patterns were seen in wind speed data, other than an apparent drop in wind during June. Raw meteorlogical data are summarized in Table S1.

**Figure 3:**
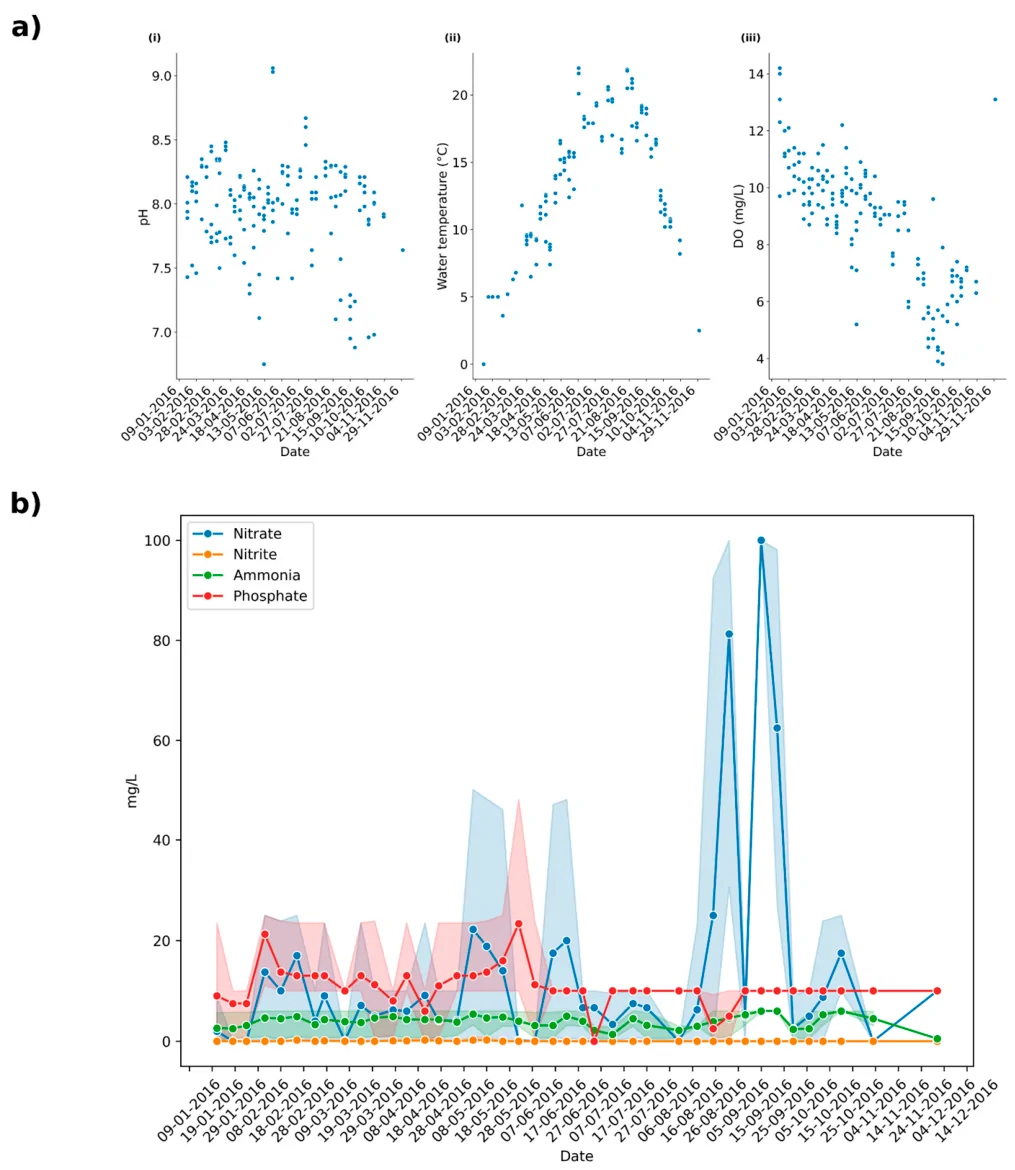
Plots of (**a**) (**i**) pH, (**ii**) dissolved oxygen and (**iii**) water temperature in lake samples and (**b**) mean nutrient concentrations (nitrate, nitrite and phosphate; mg/L) over time, with shading showing 95% percentage intervals associated with multiple sites.

Water temperatures across all samples ranged from 0 °C in the winter to a maximum of 22 °C recorded in June, resulting in a mean value of 12.1 ± 5.5 °C, with no significant difference determined among the five sites. Plots of water temperature alongside dissolved oxygen (DO) and water pH are illustrated in Figure 2. Overall, the trend in temperature was found to follow a quadratic expression (y = −0.0006x^2^ + 49.707x − 1 × 10^6^; r^2^ = 0.8233). DO values ranged from a maximum of 14.4 mg/L, recorded in the coldest months, down to a minimum of 3.5 mg/L in September. A clear drop in DO values was visible throughout July to October (Figure 2), as well as a slight drop in mid-May. Water pH was found to remain at a fairly constant weak alkaline level throughout the study, with a mean value of 7.96 ± 0.36. The highest value reached was 9.06 on 25 May 2016 at Site 17, with the lowest recorded just two weeks earlier on 12 May 2016 at Site 5. In terms of site-specific observations, lower mean pHs of 7.70 and 7.87 were determined at the shallow SW corner of the lake in Sites 5 and 7, respectively, whereas the open-lake mean pHs were 8.07, 8.11 and 8.06 for Sites 11, 14 and 17, respectively (Table S3). Nutrient concentrations were seen to vary throughout the year (Figure 3), with the most notable increases seen in nitrate levels, occurring towards the end of the summer period in September, reaching a maximum of 100 mg/L. Phosphate concentrations remained relatively consistent throughout the year, with one minor peak observed in mid-May at 50 mg/L, but with the majority of results between 10 mg/L and 25 mg/L. Nitrite mostly remained undetected, with ammonia detected throughout the year between 0.5 mg/L and 6 mg/L and no clear peaks in concentration observed. Given the consistency in phosphate throughout the year, N:P ratios mirrored the nitrate concentrations determined.

### 2.2. Cyanobacteria Presence

**Figure 4:**
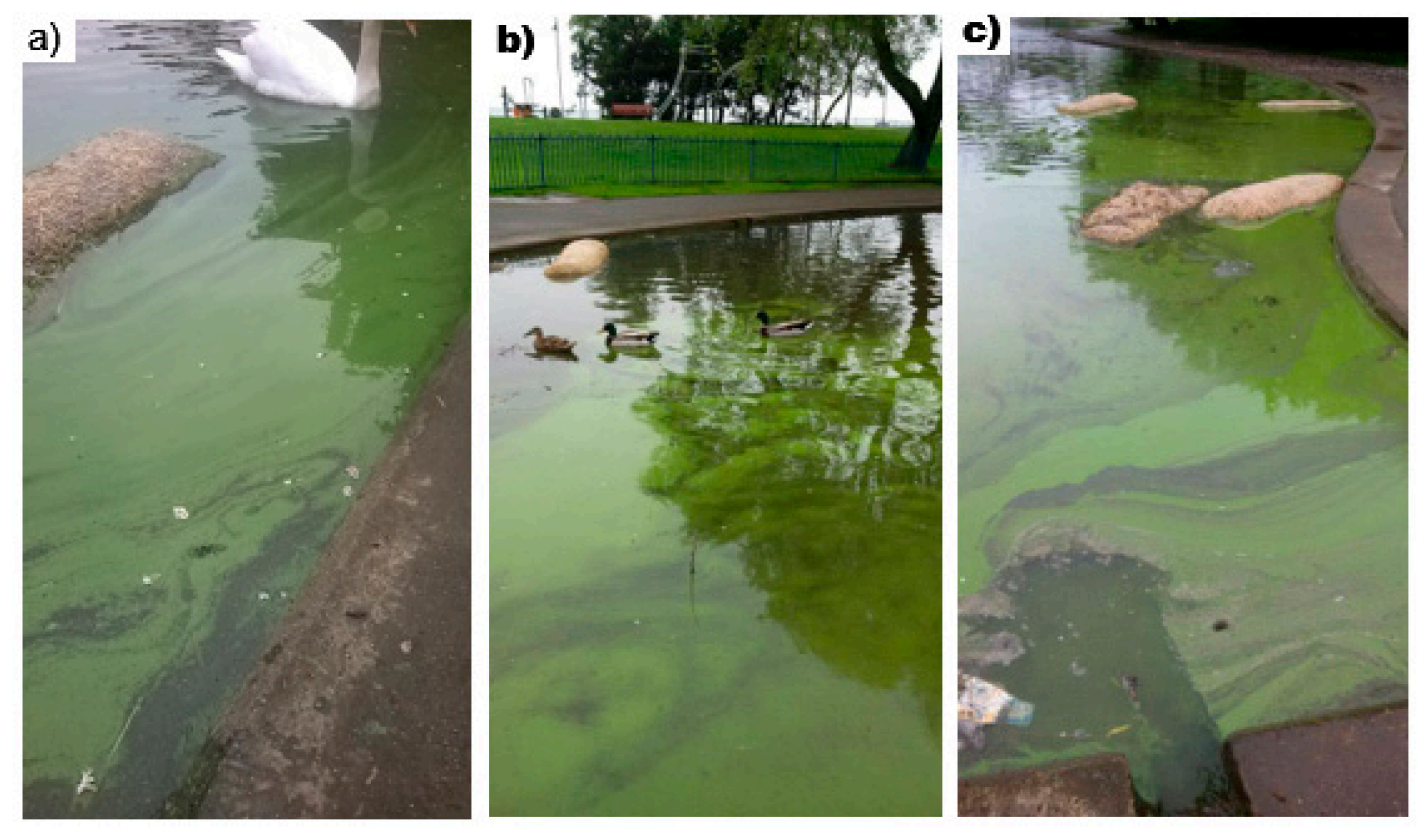
Images of parts of Portishead Lake on 15 September 2016, taken at sampling points: (**a**) Site 17, (**b**) Site 11, (**c**) Site 14.

Four out of five sites sampled in January contained cyanobacterial cells, notably at the SW corner (Sites 5 and 7). A large proportion of the lake contained surface ice, yet the cells were clearly visible under the ice. A week later, the ice had melted and less algal matter was visible, until completely disappearing by 5 February 2016. Some algal cells were seen at low apparent densities during the end of February, but the first significant bloom was observed at Site 17 in the SE corner on 26 February 2016. No further blooms were seen until the beginning of May, when a sudden proliferation was observed. Sampling officers and citizen scientists deemed the bloom to be obvious, with a strong sulphorous smell in evidence at Site 5 on 12 May 2016. Figure 4 shows images of the blooms at Sites 11, 14 and 17 during May. Visual blooms continued until mid-June, with only a small bloom seen at Site 11 (NW corner) on 22 June 2016. The next visually evident bloom was seen in mid-August at multiple sites, which continued periodically through August and September. No further cyanobacterial cells were seen visually by sampling officers for the rest of the year. Table S2 summarizes brief sampling notes from samples throughout the year at each of the five sites. Across all the samples, the presence of cyanobacterial scum was noted on six occasions.

#### 2.2.1. Microscopy

**Figure 5:**
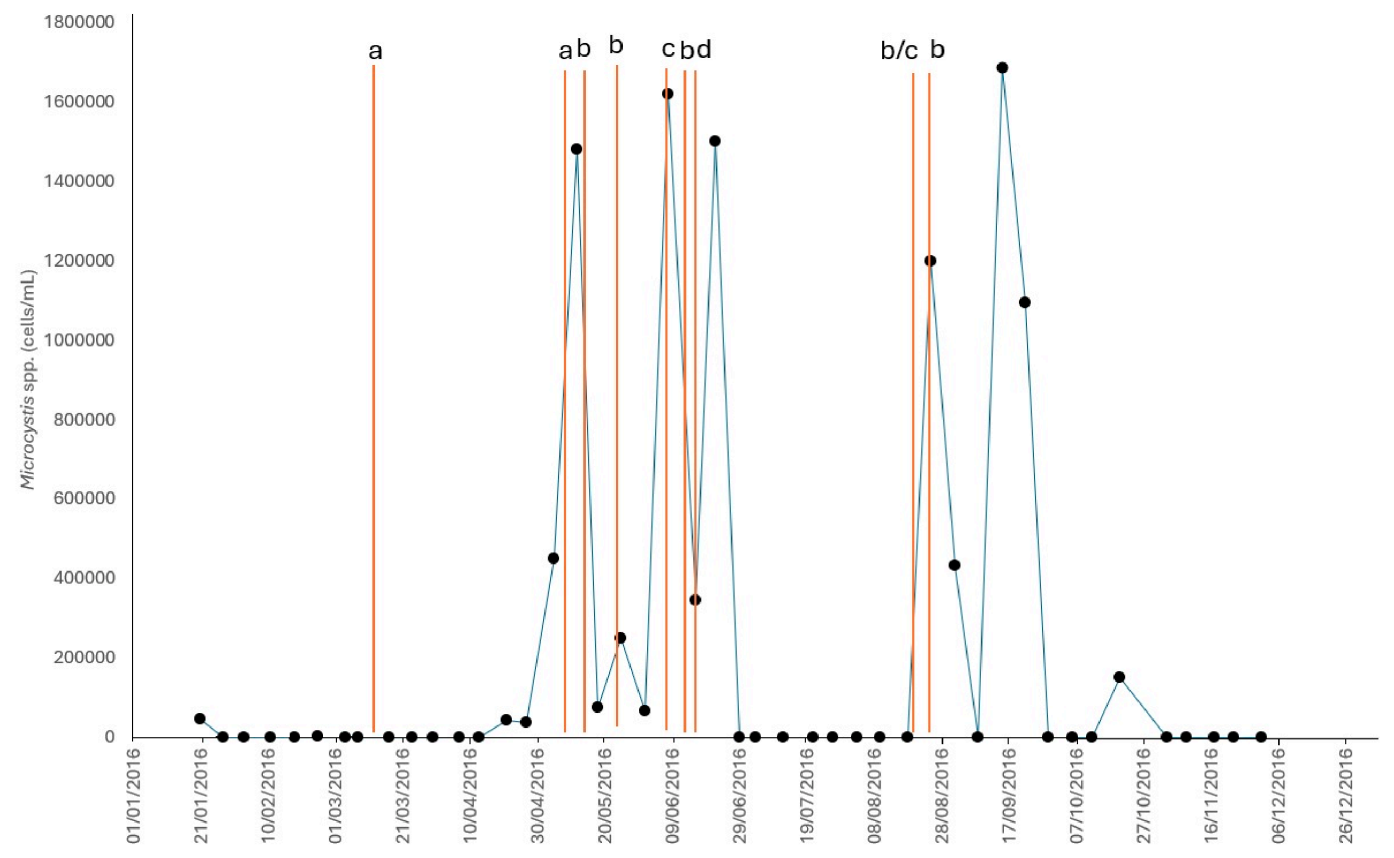
*Microcystis* spp., cell densities (mean across five sites at each time point; cells/mL) as determined by light microscopy, highlighting timing and nature of intervention conducted (a = addition of barley straw bales, b = sluice opening to allow water to flow out of the lake, c = debris removed, d = ultrasound equipment installed).

Water samples were analyzed by light microscopy for cyanobacterial species, and where recognized and detected, cell densities were enumerated. Figure 5 plots the cell densities quantified in water samples throughout the study period, showing the mean values calculated where densities were enumerated in more than one site in any given week. Analysis revealed the cyanobacteria present were exclusively *Microcystis* spp., thereby highlighting the potential risk for microcystins present within blooms.

Whilst Microcystis cells were quantified in the first week’s samples from under ice, densities were low, reaching 60,000 cells/mL in the main lake (Site 5) and 120,000 cells/mL at Site 7 in the paddling area. Overall, several major peaks in bloom density were seen throughout the year, specifically in samples taken on 12 May 2016 (1.48 × 10^6^ cells/mL), 8 June 2016 (2.20 × 10^6^ cells/mL), 22 June 2016 (2.80 × 10^6^ cells/mL), 25 August 2016 (1.20 × 10^6^ cells/mL) and 15 September 2016 (5.6 × 10^6^ cells/mL). In total, cell densities exceeded the 20,000 cells/mL guidance threshold in 26 samples, taken on 15 different weeks (Table S2). With a few exceptions, the majority of these non-compliant samples were associated with single sites within the lake, although the most affected site was not constant throughout the year. The notable exception was 15 September 2016, when high cell densities were quantified at four out of five of the sampling sites, with Site 5 exhibiting the highest density across the whole year.

#### 2.2.2. IOLight Portable Microscopy

**Figure 6:**
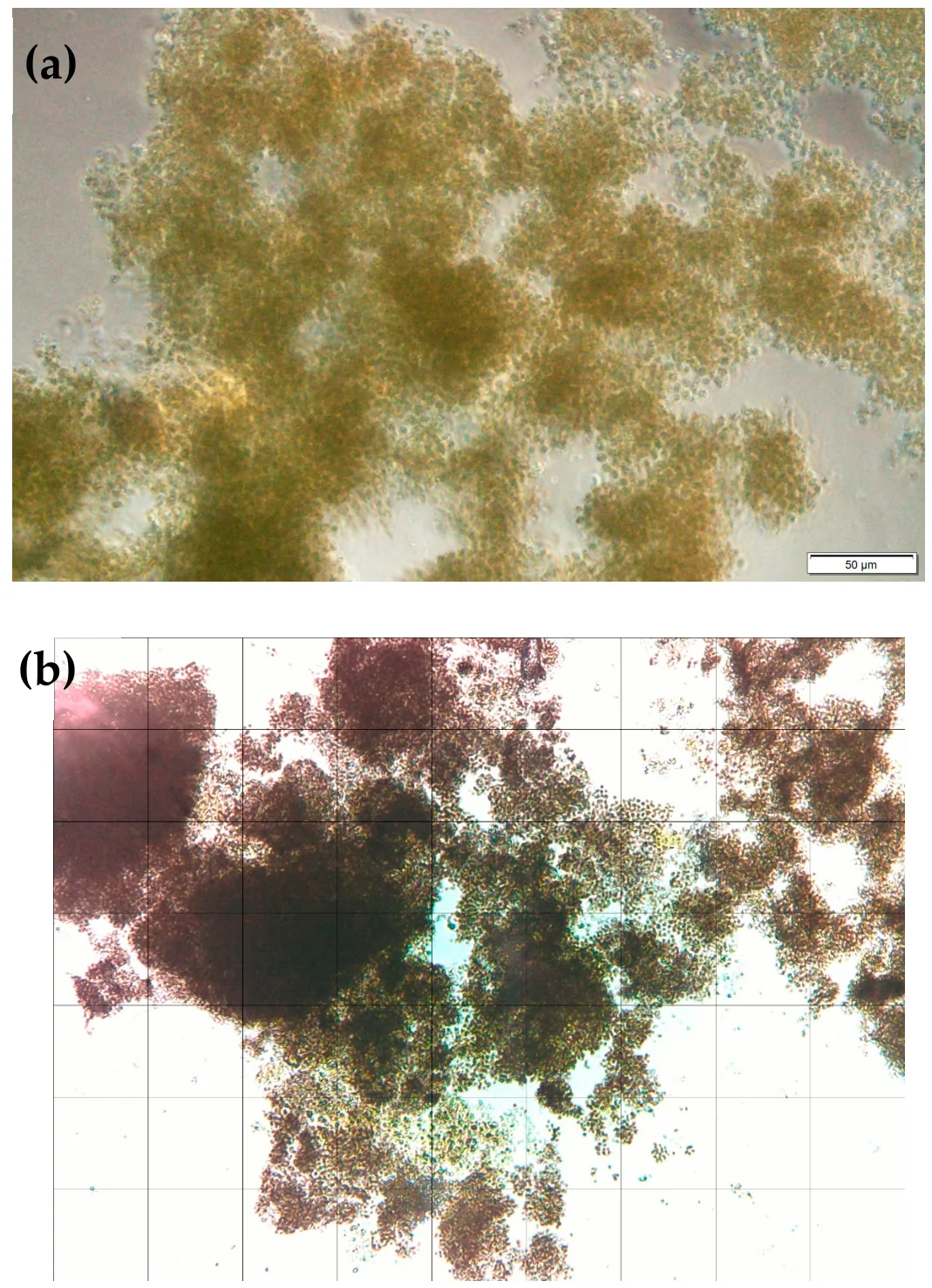
Micrographs of environmental water samples containing *Microcystis* spp. using: (**a**) ×25 magnification laboratory image using inverted microscope; (**b**) IOLight ×10 magnification portable compact cell imager. Grids are 0.1 mm × 0.1 mm.

The IOLight portable microscope became available commercially after the end of the study. However, archived water samples including *Microcystis* cultures and environmental samples were assessed. Figure 6 illustrates the comparison between micrographs obtained using the IOLight microscope and the laboratory microscope. The images obtained using the IOLight scope at ×10 magnification were found to provide effective visualization of the Microcystis colonies, with the images clear enough to facilitate cell density measurements of water samples without use of the ×25 magnification laboratory microscope.

### 2.3. Cyanotoxin Analysis

#### 2.3.1. Intracellular MCs

**Figure 7:**
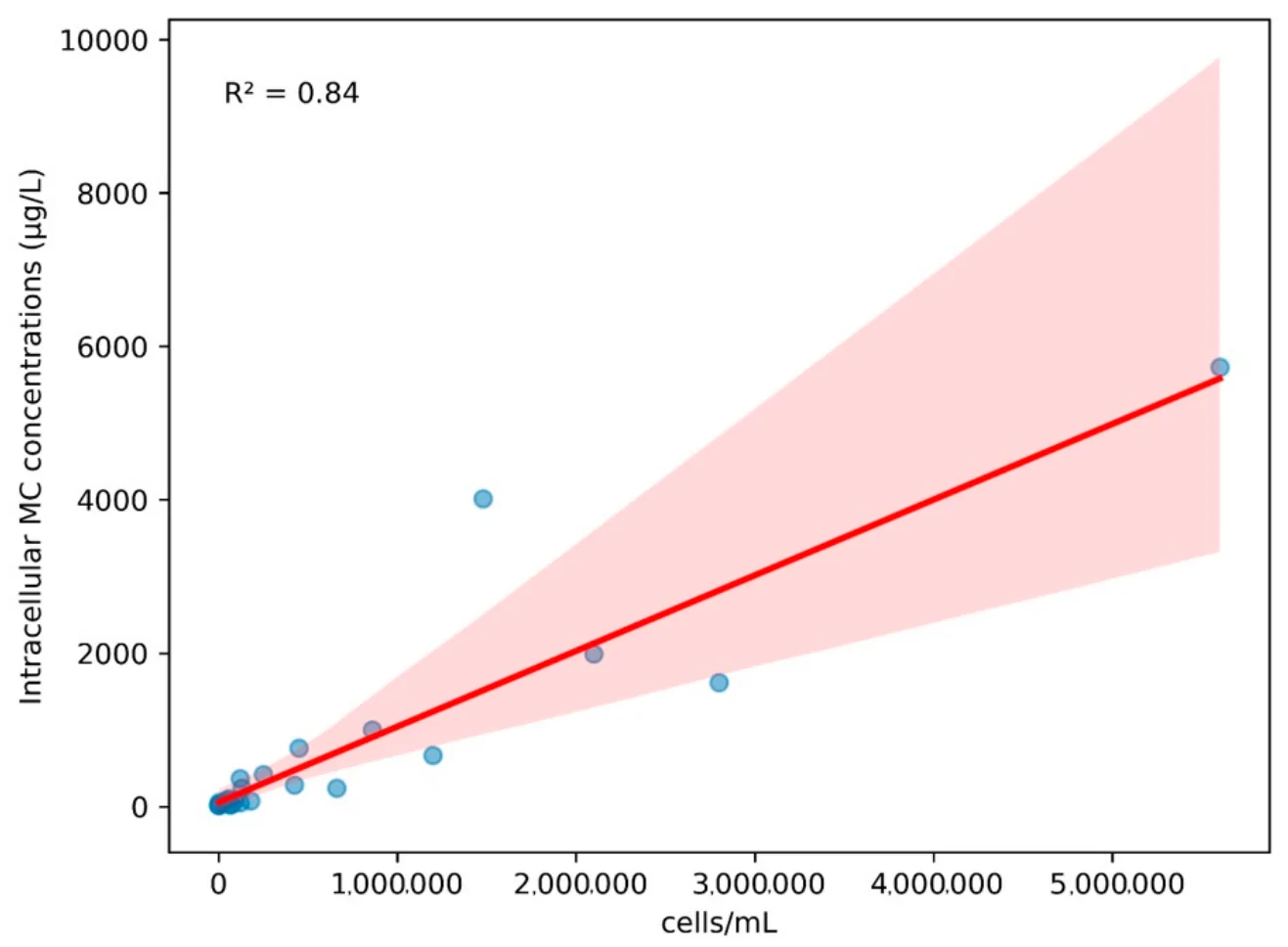
Graph plotting linear regression between *Microcystis* spp. cell densities (cells/mL) and total intracellular MC concentrations (µg/L), with red shaded area highlighting 95% confidence of the regression.

**Figure 8:**
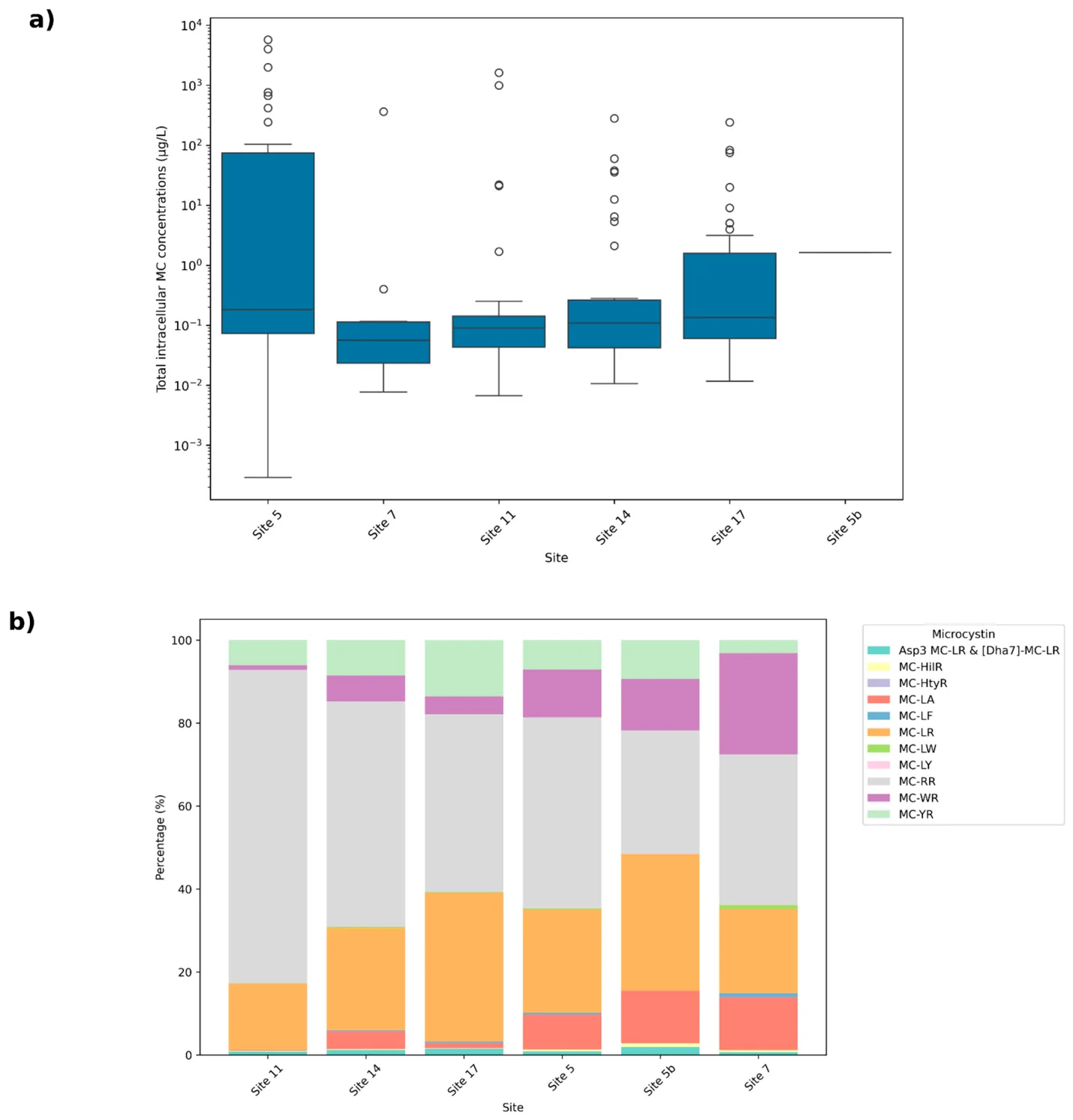
(**a**) Box-and-whisker plot of total MC concentrations at each sampling site. Horizontal lines represent the median, boxes represent the 1st and 3rd quartiles, and dots represent outliers. (**b**) Bar chart showing toxin profiles in terms of proportion of MC analogue compared to total MC concentrations for each analogue at each of the sampling sites.

**Figure 9:**
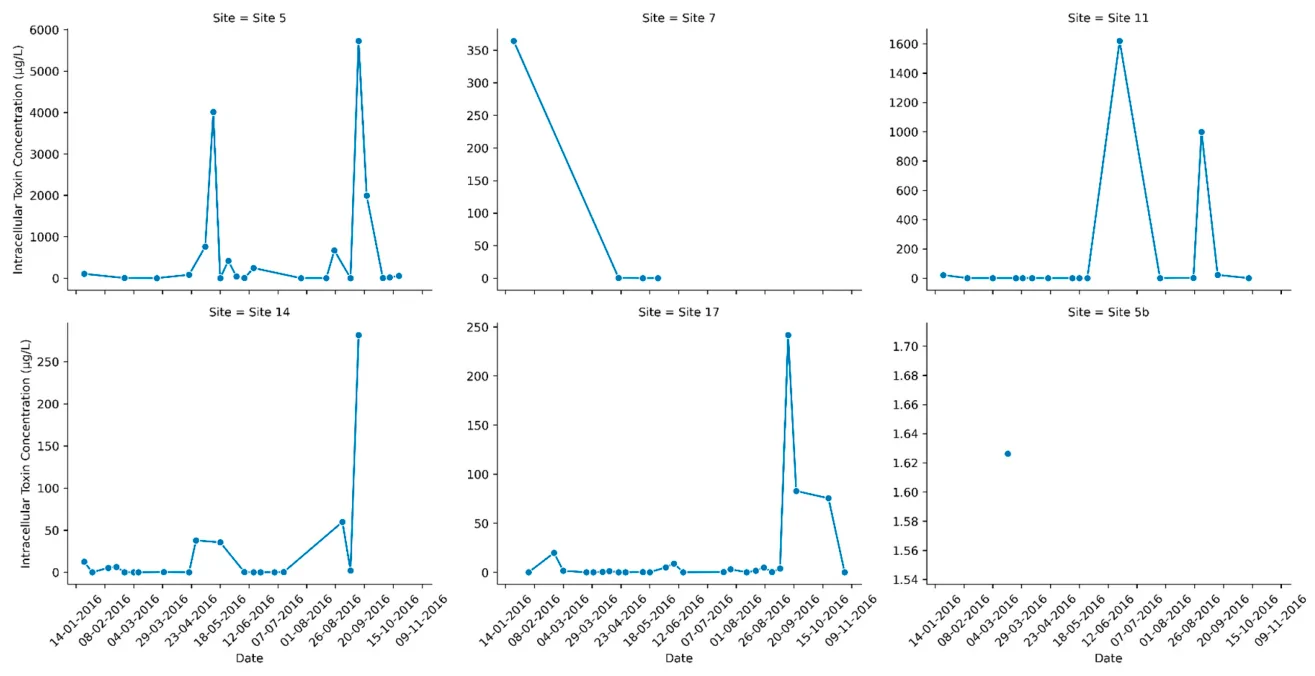
Plot of total (summed) MC concentrations (µg/L) at each sampling site throughout the study.

Figure 7 illustrates a close correlation between *Microcystis* spp. cell densities and total intracellular toxin concentrations quantified by LC-MS/MS. Figure 8a and Figure 9 show the distribution of intracellular MC concentrations at each site and the variability in total MC concentrations determined throughout the year at each site. Data illustrated notable differences in toxin levels between the sites. Table S4 tabulates all the values quantified throughout the study period. The maximum summed MC concentration reached was 5728 µg/L at Site 5 on 15 September 2016, followed by 4012 µg/L on 12 May 2016, also at Site 5. Site 11 was found to contain the next highest concentrations, with maxima on 22 June 2016 and 1 September at 1620 µg/L and 999 µg/L, respectively. Sites 14 and 17 contained low total MC concentrations, with 282 µg/L and 242 µg/L reached at the same time on 15 June 2016. Site 7 showed very low levels of toxins throughout the study, with the exception of the first week when 365 µg/L total MCs were quantified in the water sampled from under ice. After this point, the toxins did not exceed 1 µg/L at any time (Table S4).

The quantitation of individual MC analogues by LC-MS/MS enabled the determination of toxin profiles across all five sampling sites throughout the year. A large range of analogues were detected, particularly in samples containing high total toxin concentrations. Some of the highest concentration samples contained the majority of analogues incorporated into the LC-MS/MS method, with the Site 5 sample from 22 September containing all analytes (Table S4). Figure 8b illustrates the toxin profiles across each individual site and shows fairly consistent profiles between the sites, with the exception of MC-RR, which appears to dominate Site 11. Other than this, the most commonly occurring analogues were MC-LR (31% ± 17%), MC-RR (41% ± 23%), MC-WR (20% ± 16%) and MC-YR (15% ± 16%). Lower relative proportions were detected for MC-LA and MC-LF, with small levels on average for MC-LW, dm-C-LR and MC-HilR and only occasional trace concentrations of MC-HtyR and MC-LY detected.

#### 2.3.2. Extracellular MCs

**Figure 10:**
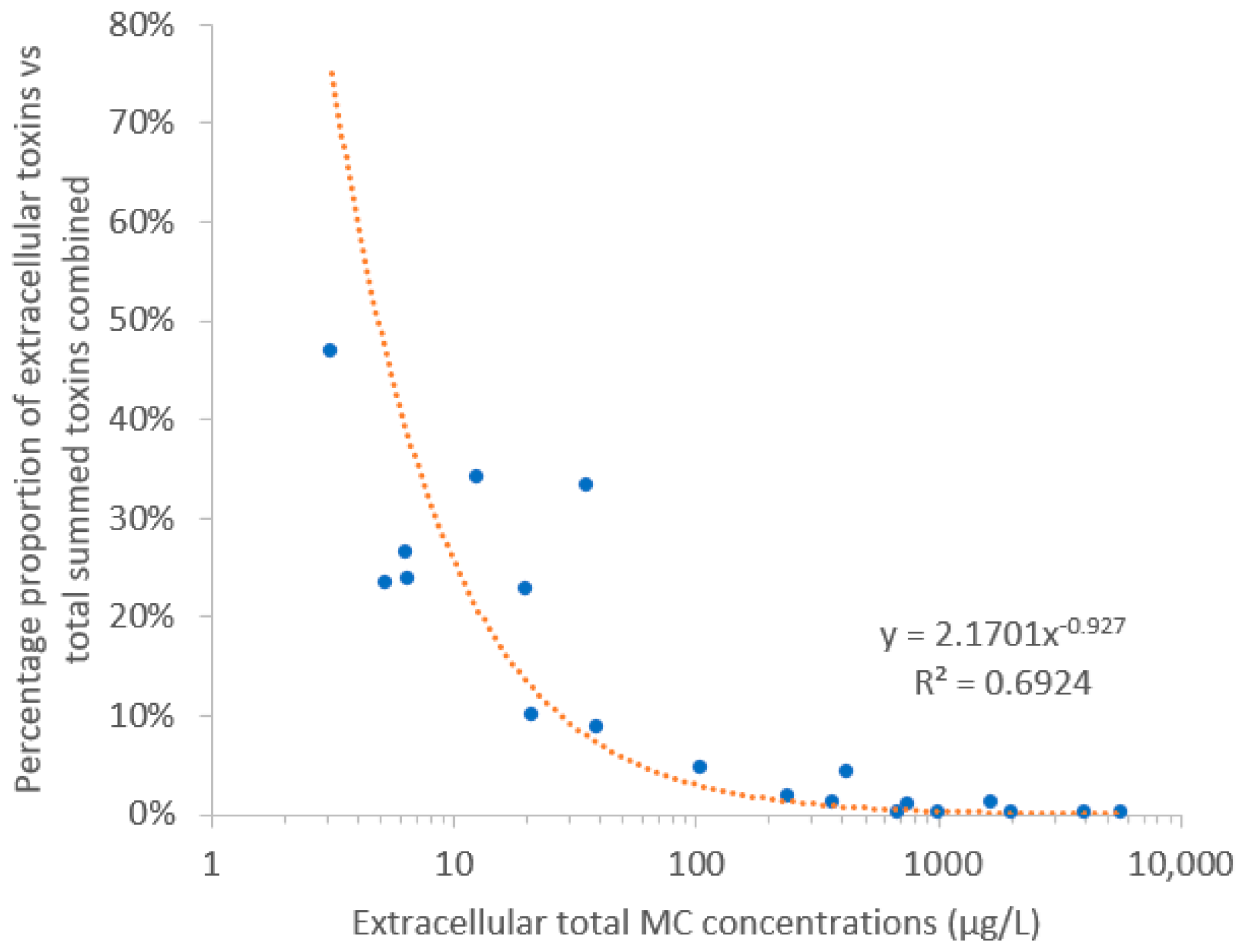
Graph highlighting the relationship between the percentage of extracellular toxins out of total (extracellular and intracellular combined) and the actual total extracellular MC concentrations. Graph shows correlation coefficient (r^2^) and curve equation.

Lower extracellular MC concentrations were quantified in the water samples as compared to the intracellular content. Total MCs reached a maximum of 21.5 µg/L from Site 11 taken on 22 June 2016, when 1620 µg/L total intracellular MCs were quantified. The proportion of extracellular to intracellular toxins was seen to be generally very low, when high concentrations of MCs were quantified in the more prolific blooms, with the majority of samples containing total intracellular concentrations over 100 µg/L exhibiting <2% as extracellular toxins. The percentage of extracellular toxins did increase, however, at lower total concentrations as illustrated in Figure 10, with a power regression described by y = 2.170x^−0.927^ (R^2^ = 0.692).

### 2.4. Statistical Analysis

**Figure 11:**
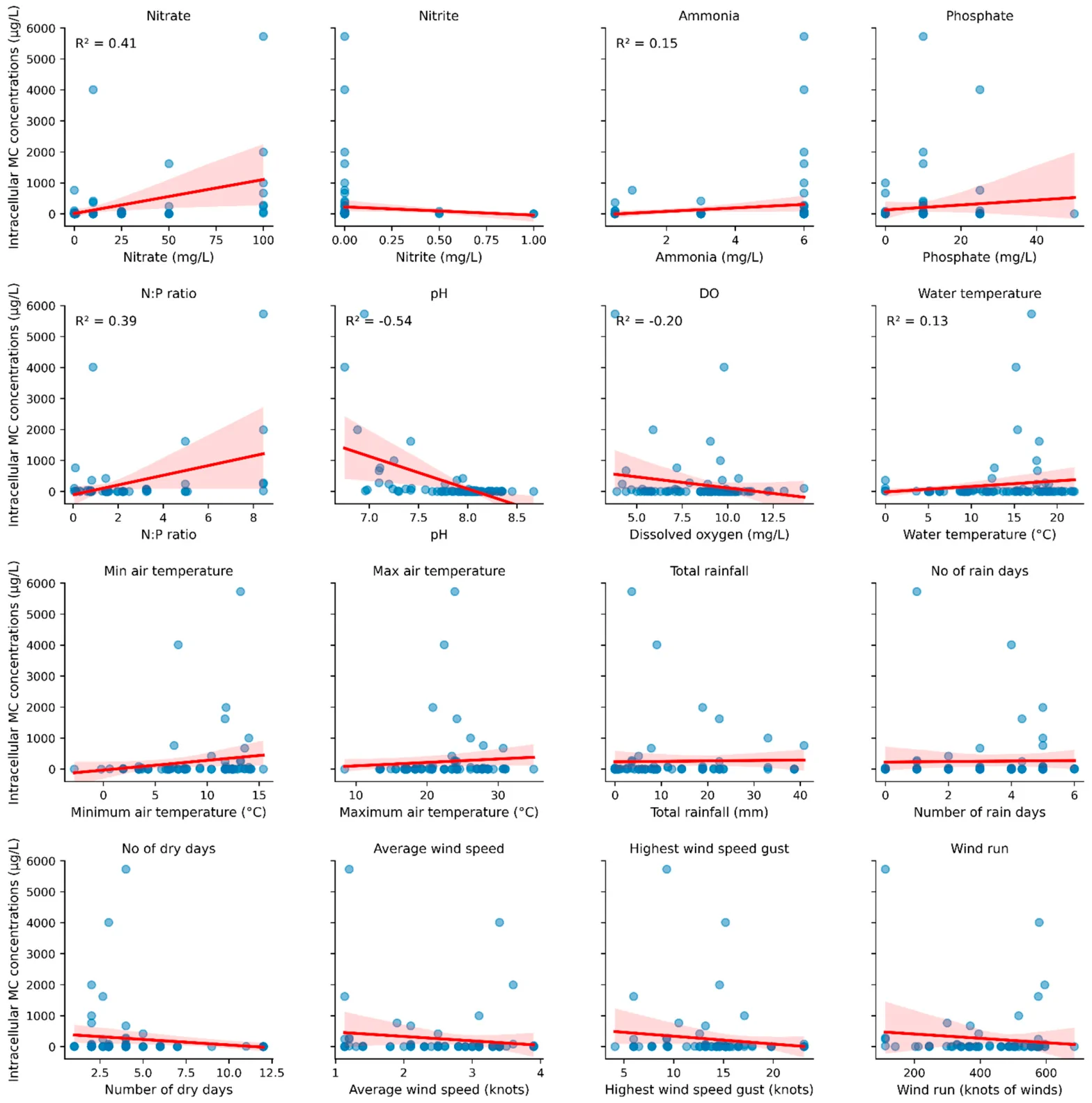
Facetted plot of Intracellular MC concentrations vs. environmental and nutrient variables. Linear regressions are represented by red lines, and shaded areas are the associated 95% confidence intervals. All regressions >0.1 are displayed in facets.

**Figure 12:**
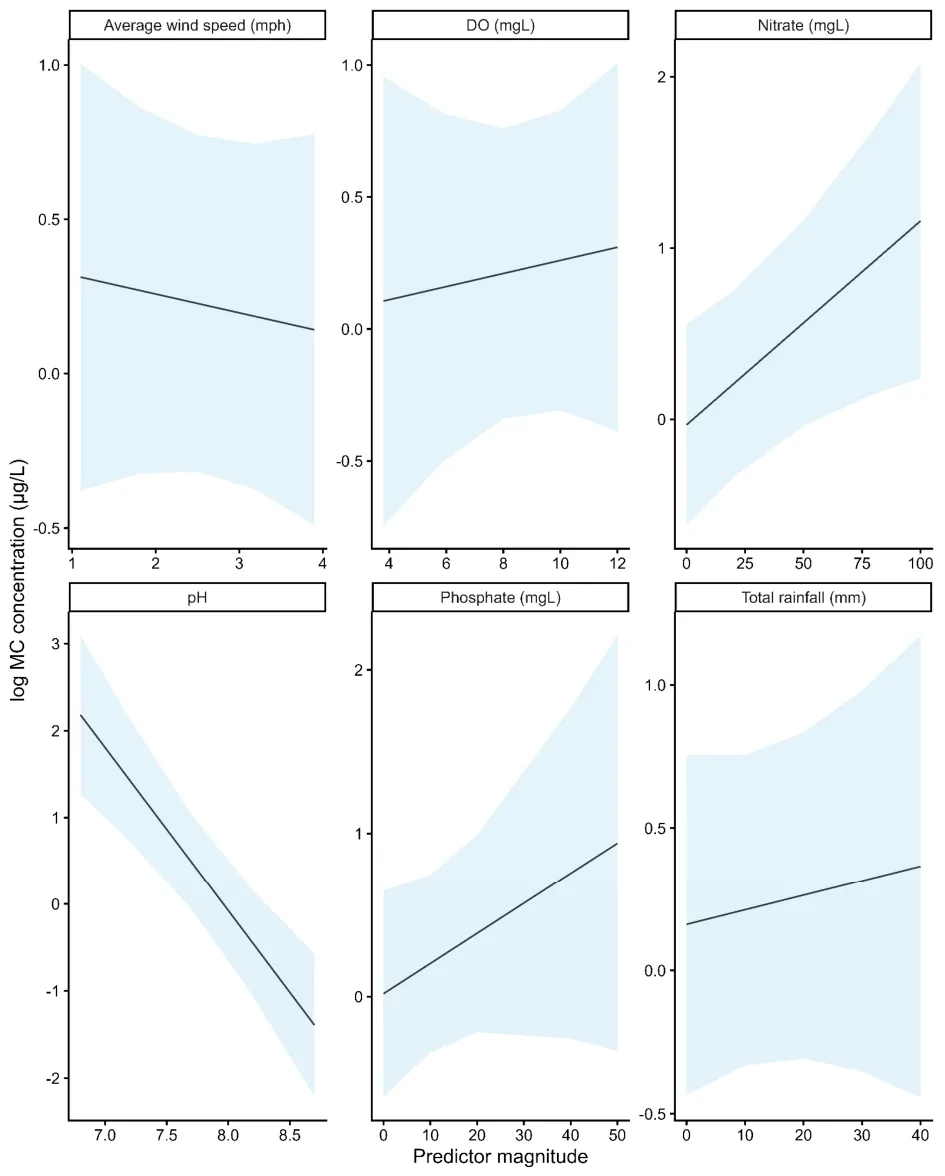
Partial-effect plots for each variable included in the most parsimonious linear mixed-effect model. The x-axis is variable magnitude, and the y-axis is log intracellular MC concentrations.

**Figure 13:**
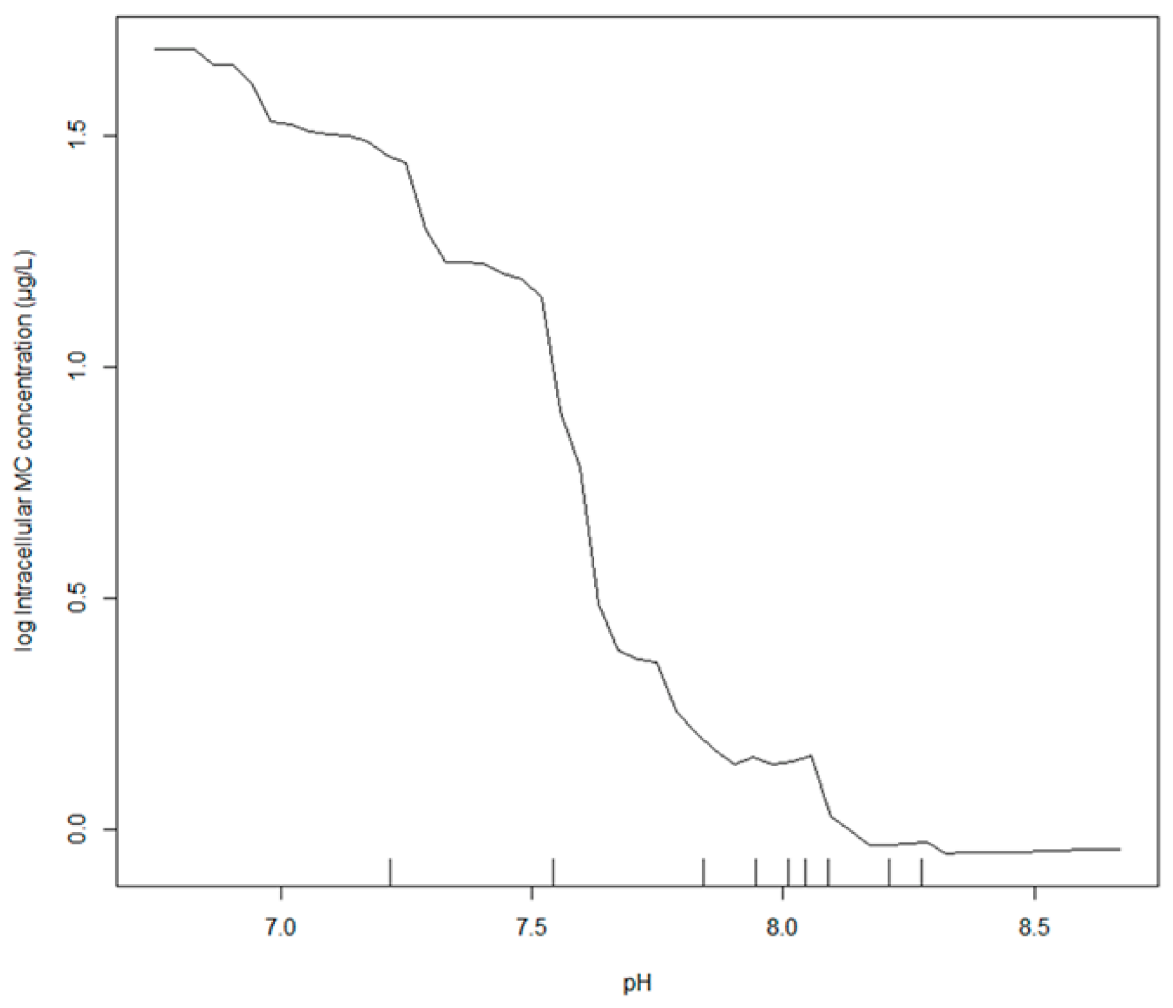
Random Forest partial dependence plot of pH vs. log intracellular MC concentrations (µg/L). Vertical marks on x-axis represent distribution of pH data used to build the Random Forest model.

Univariate linear relationships among environmental variables and intracellular MC concentrations are shown in Figure 11. To assess which of these environmental drivers were most associated with intracellular MC concentrations, linear mixed-effect models were built and simplified to achieve a parsimonious fit. Intracellular MC concentrations were log-transformed before analysis to reduce the chance of violating our Gaussian errors assumption. One or both variables found to be co-linear (with a pairwise correlation exceeding 0.5) were removed prior to analysis [39], and AIC and BIC were used to compare model fits. Variables that did not increase model fit were also removed. Site was included in all models as a random effect. The full output and the residual plot of the most parsimonious model (that with the lowest AIC and BIC when variables with negligible effects were removed stepwise) can be found in Table S6 and Figure S2, respectively. To summarize, the model fit suggested that decreasing pH had the largest effect on intracellular MC concentrations (*p* = <0.000001, estimate = −1.88). Increasing nitrate concentrations also had a statistically significant effect on intracellular MC concentrations (*p* = 0.0171, estimate = 0.011). With phosphate concentrations consistent throughout the year, nitrate concentrations and N:P ratios were highly correlated. To analyze the effect of sites on MC concentrations, a second series of models were created. Firstly, an ANOVA showed site as being significantly related to MC concentrations (*p* < 0.001). Then, a parsimonious linear model (same variables as above) was fitted to assess individual sites. Sites 5 and 7 had significantly different concentrations from the reference site (Site 11). Full ANOVA and linear model outputs can be found in Figure S5. Tukey’s post hoc analysis of means was then conducted between each pair of sites that highlighted Sites 5 and 7 as being significantly different from each other and some other sites. Full output can be found in Figure S6. These models showed Site 5 as having significantly higher concentrations and Site 7 as having significantly lower concentrations. This was confirmed visually by analyzing the random effects from the linear mixed-effect model (Figure S3). Partial-effect plots for variables included in the model can be seen in Figure 12. This analysis was confirmed using linear regression (Figure 11 displays an r^2^ of 0.41 and −0.54 for nitrate concentrations and pH, respectively). To confirm the conclusion that lowering pH and increasing nitrates were the main effects on intracellular MC concentrations, a parsimonious Random Forest model was constructed with 2000 trees and three variables per split and explained ~47% of the variability in the dataset (Figure 13). The model incorporated all variables that increased the percentage variability explained by the model. The Random Forest model agreed that pH and nitrate concertation were the most important variables (47.06% and 24.65% increase in mean square error (%IncMSE), respectively, with the full importance plot given in Figure S4). A partial dependence plot of pH vs. log intracellular MC concentrations is displayed in Figure 13 and confirms the inverse relationship between MC concentration and pH. All analyses were performed in R version 4.4.1. Linear mixed-effect models were created using lme4 package [40]; partial-effect plots were made using the effects package [41,42]. The Random Forest model was built using the Random Forest package [43].

### 2.5. Intervention

**Figure 14:**
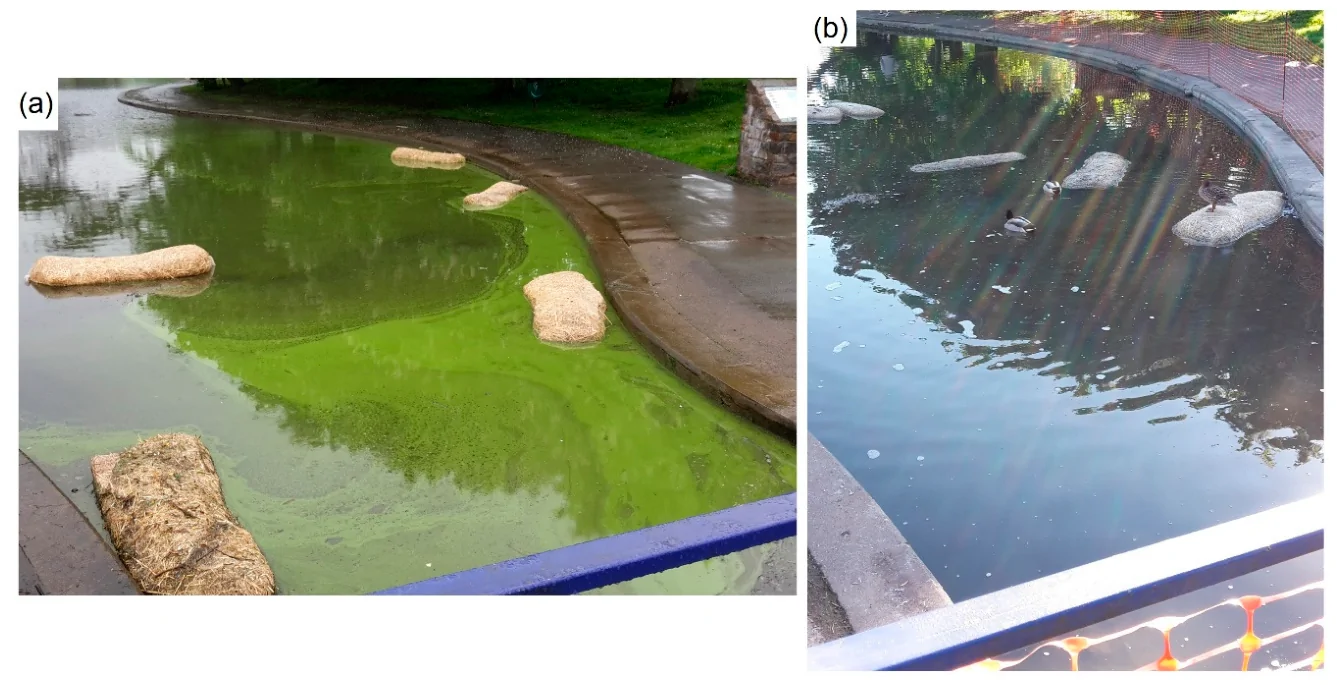
Site 5 in May showing presence of barley straw bales added by local authority workers on (**a**) 10 May (**b**); 16 May.

Some intervention measures were conducted during the study period, as summarized in Table 1. New barley straw bales were added across the lake (n = 60) in mid-March, two weeks after there were some indications of blooms starting two weeks earlier. The bales were concentrated particularly at the corners of the lake where cyanobacterial scums were anticipated to accumulate following the effects of wind. No further intervention was conducted until the first large blooms were seen in early May, at which point the sluice from the main lake was opened for a few hours on three separate occasions during the month, and a further occasion in June, to allow water flow out of the lake. On two occasions, vegetation and litter debris were cleared from sections of the lake to aid within-lake water flow. Finally, in mid-June, solar-powered ultrasound equipment was installed near the centre of the lake (Figure 2) in order to attempt to disrupt the growth of cyanobacterial blooms. Figure 14 illustrates the barley straw bales added to the water at Site 5 on 10 May 2016 clearly containing cyanobacteria and the subsequent visible lack of cynobacteria six days later once water flow had been allowed on two occasions. Figure 4 marks the intervention measures applied on the graph of mean weekly *Microcystis* spp. cell densities. Out of the measures applied, the opening of water sluices to increase the overall water flow in the lake was the only measure that appeared to be followed by a reduction in cyanobacterial cell densities within the subsequent week.

## 3. Discussion

### 3.1. Cyanobacteria and Microcystin Presence

Cyanobacterial growth was identified within the waters of Portishead Marine Lake during the periodic presence of extensive visual blooms, with *Microcystis* spp. evidenced by light microscopy and with scum formation reported. Cell density data showed four periods of highest prevalence, during early May, late June, late August and mid-September. Consequently, the highest cell densities of *Microsystis* were measured during the warmer months of the year, when water temperatures rose above 15 °C. This is expected given the optimal growth of many cyanobacteria species at higher temperatures [44,45,46,47] and reports of positive correlations between air and water temperatures with cyanobacterial cell densities [48,49,50,51,52,53,54]. In this study, whilst there was a low statistical correlation between water/air temperatures and toxin production (expressed as intracellular MC concentrations), for samples containing *Microcystis* cells, there were clear high toxin concentrations in water samples taken during times of higher temperature. The one exception to this was the findings of *Microcystis* cells under ice in January 2016 and the quantitation of MCs from the extracted cellular material, as reported by others [55].

DO levels were found to start dropping slowly at the time of the first major bloom in early May, before falling markedly towards later summer from August onwards. This is understandable given the evidence for the microbial decomposition of cyanobacterial biomass depleting oxygen within lake waters [48,49,50,56]. In this study, pH did not drop below neutral levels, with a mean pH of 7.96 ± 0.36 across the entire lake, and with lower mean pH determined at Sites 5 and 7 (7.70 ± 0.43, 7.87 ± 0.31, respectively) in comparison with Sites 11, 14 and 17 (8.07 ± 0.28, 8.11 ± 0.32 and 8.06 ± 0.29, respectively). These observations therefore fit with the general consensus that pH levels between 6 and 9 are most favourable for cyanobacterial growth [57,58,59]. The negative correlation determined, however, between intracellular toxin concentrations and pH was still an interesting finding. The majority of previous studies describe a positive correlation between these two parameters. Increases in water column pH during *Microcystis* spp. blooms have been reported in multiple water bodies throughout the world with cyanobacteria growing at alkaline pH [60,61,62,63,64], with optimal *Microcystis* growth conditions observed at pH 8.5. pH increases are also reported in cell cultures of *Microcystis* sp., with positive correlations between pH and cellular growth [65] as a consequence of photosynthetic consumption of carbon dioxide [66,67], resulting in a competitive advantage to cyanobacterial growth due to cyanobacterial carbon acquisition [68,69]. Not all studies report similar results, however. The highest MC-RR production was found to occur in cultures of *M. viridis* at both low (7.0) and high pH (9.2) levels, with significantly lower toxin production at pH 7.8 and 8.6 [70]. Other reports of cyanobacteria blooms leading to lower water pH do exist, however [71]. Cyanobacteria densities and MC concentrations in Hulun Lake, China, were, for example, negatively correlated with pH [72]. Ref. [73] also showed pH reduction from 10 to 7.50 coinciding with cyanobacterial cell density increases in a lake in Bangladesh, followed by subsequent pH increases back to 10 as the bloom subsided. Overall, therefore, the relationship between pH and toxin production in this study still fits with those patterns described in several previous studies. The influence of human-induced eutrophication and climate change on cyanobacterial bloom proliferation is well described in both marine and freshwater systems, with cyanobacteria dominance recognized as freshwater bodies are becoming more nutrient-enriched [50,74,75], and some studies evidencing stronger correlations between cyanotoxin concentrations and nutrients than between toxins and water temperatures [76]. Human activities including runoff from agricultural land, sewage inputs and industrial effluents are all known to trigger cyanobacterial growth and ultimately impact recreational water quality [77]. Variability is exhibited, however, between different cyanobacterial species in terms of their response to N and P concentrations and temperature [78,79]. Cyanobacterial blooms themselves are also known to change both the chemical and physical properties of water bodies as well as releasing N and P into the water during cell death, further exacerbating eutrophication within the affected lake [80]. Such changes are more likely to be expected in shallow water bodies, such as the Portishead marine lake [81]. Whilst phosphate concentrations remained relatively consistent throughout the year in this study, there were clear increases in nitrate concentrations during each of the four bloom periods, especially during the later bloom maxima in August and September, resulting in the observed positive correlation between nitrate and MC concentrations, as well as N:P ratios and toxin levels. This correlation fits with reports of the highest levels of toxins in *Microcystis* strains when grown in media with high nitrogen levels [82,83,84,85].

Although nutrient measurements were conducted in this study using portable testing kits suitable for use by citizen scientists, the data still evidenced N:P ratios between 0.05:1 and 8.5:1. Together with nitrate and phosphate concentrations reaching maximum values of 100 mg/L and 50 mg/L, respectively, there are indications of a highly eutrophic, nitrogen-limited water body [86]. Consequently, it was clear that the study lake had been affected by phosphorous accumulation in the ecosystem. Given the absence of agricultural land, industry and sewage discharge outlets in the vicinity, it is likely that the large numbers of birds in and around the lake were responsible for these nutrient inputs (Figure S1c).

Results also demonstrated clear intra-lake differences in cyanobacteria and microcystin levels between sampling sites. ANOVA results confirmed site as a statistical driver of MC concentrations. Median and maximum total toxin concentrations reached the highest levels at Site 5, situated at the southern end of the lake at a topographical restriction. Site 5 also had significantly higher MC concentrations than other sites. Whilst the prevailing wind direction across the majority of areas of the UK lies between west and south, significant variation is seen in this regard as a consequence of local and regional topography as well as season, with parts of the southwest of England having a mean annual wind direction of northwesterly [87]. Consequently, the restriction at Site 5 is likely to exacerbate the accumulation of wind-driven cyanobacterial cells, especially when scum formation is experienced. Therefore, a combination of wind effects and local lake topography is most likely a major cause of the highest toxin concentrations quantified at Site 5. In tandem, Site 7 showed significantly lower MC concentrations than other sites. This is likely a result of the constriction after Site 5 that effectively cuts off the site from the rest of the water body, especially during periods of low water level.

The results from this study can be compared with data obtained in other UK and European freshwater bodies [8]. A UK study of three freshwater reservoirs in 2019 revealed a high diversity in cyanobacterial metabolites including microcystins, with large inter-lake differences in toxin concentrations, but with concentrations notably lower and all below the WHO recreational guideline limit, than those reported here [88]. The same study also reported maximum toxin levels at the end of the summer (September), as opposed to the multiple peaks in bloom densities in this study [88]. Ref. [20] reported highly variable cyanobacteria cell densities and microcystin concentrations across multiple freshwater bodies in England during the same year. Although multiple different cyanobacterial genera were identified across wide-ranging waterbody types, the highest cell densities and toxin concentrations were notable in May, July, August and September, thereby showing similar patterns to the maximum *Microcystis* spp. cell densities reported in Portishead Marine Lake [20]. A major study of multiple freshwater lakes in Belgium showed a similar range of toxin levels quantified, with total microcystin concentrations ranging from 0.11 µg/L to 27,998 µg/L, albeit produced by a wide range of cyanobacterial genera [89]. A large study of cyanobacterial blooms and cyanotoxins in 92 lakes across Central Europe during the summer of 2015 showed significant differences in cyanobacterial species and toxin composition between warm lakes and temperate water bodies, with microcystin concentrations correlating significantly with cyanobacterial biomass in the cooler lakes [76].

### 3.2. Microcystins

MC concentrations determined in this study were higher than the average values reported in some previous studies [11,90,91], including a large study focused on the determination of cyanotoxins in six eutrophic lakes in China [72], although they were similar to those quantified in 137 water samples taken from natural lakes, reservoirs and artificial lakes throughout England during the same year [20]. In that study, total MCs quantified above the 24 µg/L WHO limit ranged from that limit to a maximum of 42,724 µg/L, with all except three samples returning total MC concentrations below 1000 µg/L. Toxin concentrations were also found to peak in May, August and September, evidencing similar seasonality in Portishead Lake in comparison with other English water bodies. Toxin concentrations quantified at the highest concentrations in this study were those associated with clear visual scum formation, thereby explaining the very high concentrations encountered periodically. Accurate quantitation of toxins in water containing scum material is challenging given the difficulties with sampling and creating a homogenous sub-sample for toxin extraction, so these results represent a “worse-case” scenario of toxin levels from floating cellular materials during the peaks of blooms. 

A large number of MC analogues were detected and quantified, with MC-LR and MC-RR being the most commonly occurring MCs, followed by MC-WR and MC-YR. These findings agreed closely with the previous reports of English water bodies being dominated by MC-LR, MC-RR and MC-YR [20,88] as well as in Chinese eutrophic lakes [72]. Therefore, the current study reported only the presence of *Microcystis* spp. in comparison with the wider study from the same year, where twelve different cyanobacterial genera were reported including *Microcystis*, *Aphanizomenon*, *Oscillatoria*, *Gomphospaeria* and *Planktothrix* [20]. Similarly complex MC congener profiles have been reported in studies from other parts of the world including Canada, where relatively high proportions of MC-LR, MC-LA, MC-YR and MC-HtyR were reported [53] along with similarly high relative proportions of MC-LR, MC-YR and MC-RR in a survey of 98 cyanobacterial blooms from Swedish lakes and the Baltic Sea [92]. A study of multiple lakes in Belgium evidenced a dominance of MC-RR followed by MC-LR, with MC-YR significantly lower in proportion [89], with highly variable profiles also reported across 92 lakes in Central Europe [76] and evidence for decreasing MC-LR proportions in lakes at higher latitudes when assessing toxin profiles across 137 lakes throughout Europe [93]. Complex MC profiles can be quantified, but accurate toxicity equivalence factors would be required to more accurately reflect total toxicity impacts, highlighting a limitation of the use of LC-MS/MS for quantifying cyanobacterial health risks.

Whilst MCs are produced and stored within *Microcystis* cells, they can also enter the surrounding water following senescence and cell lysis. The stability of these extracellular toxins has been reported to be high at neutral water pH due to the resistance to hydrolysis and oxidation leading to post-cell-death persistence of extracellular toxins for several weeks [94]. Even during times of extremely high intracellular MC concentrations, the levels of toxins dissolved in water remained low, reaching a maximum of ~21 µg/L across the whole study. This provides strong evidence that the greatest risk to human health originates from the cyanobacterial cells, as opposed to toxins present within the water, given the potential ingestion of cellular material by waterbody users including children who paddle in the waters.

### 3.3. Monitoring and Control Measures

In this study, microscopy was utilized for the detection and enumeration of cyanobacteria and their associated cell densities. A dedicated laboratory-scale microscope provided a suitable tool for the in-house assessment of fixed samples received at the laboratory following shipment from the lake by project sampling officers. In certain instances, however, particularly in remote locations where testing laboratories are situated great distances from waterbodies, there is the need for in-field cyanobacterial monitoring or at least monitoring stations that can be operated by local authorities with limited phycological experience or even citizen scientists. In this study, the IOLight portable microscope was used for cyanobacterial detection. Images of environmental samples taken showed the applicability of this tool for remote monitoring and have been applied post-study in other freshwater monitoring areas. Furthermore, artificial intelligence (AI)-powered cell detection and enumeration tools have been developed using the same microscopes and applied in the USA for the rapid in-field assessment of cyanobacterial blooms [95]. This enabled citizen scientists to detect and enumerate harmful blooms across 36 different lakes, with taxonomic identification to genus level deemed equivalent to that of a trained phycologist. Such an approach would provide an excellent resource for local authorities and water body owners in the UK faced with significant socioeconomic impacts from harmful cyanobacterial events. Local scientists were also involved in this study with the measurement of dissolved oxygen concentrations and pH, as well as the measurement of localized weather conditions that would not have been recorded by national weather-monitoring authorities. Therefore, there were distinct benefits in combining the laboratory monitoring tools with the efforts of local scientists who were able to visit the lake on a weekly basis and take beneficial measurements, which contributed significantly to the overall study. In the wider context, however, it is difficult to determine which of these citizen science monitoring approaches is critical for the future monitoring of freshwater lakes in other regions. More data would be needed across multiple study sites in order to determine which parameters are critical for ongoing lake measurements to facilitate effective cyanobacterial risk monitoring across agencies, local authorities and citizens. Certainly, the use of commercial monitoring tools was one limitation of the work in terms of providing accurate and validated assessment methods for environmental parameter assessment.

Whilst cyanobacterial identification and cell density enumeration provide valuable data to local authorities, this information does not fully inform the overall level of risk, given the high variabilities quantified in toxin concentrations from different species, strains and geographical areas. In this study, five samples exhibited *Microcystis* cell densities above the 20,000 cells/mL threshold whilst showing intracellular MC concentrations below the WHO 24 µg/L guidance limit [77]. These samples were taken during May and June and provided interesting evidence for occasional low toxin concentrations in some areas of the lake even during high-density cyanobacterial blooms (Tables S2 and S4). Furthermore, out of the 21 samples containing total intracellular MC concentrations above 24 µg/L, all of these were samples exceeding the 20,000 cells/mL density threshold. Consequently, whilst the two guidance limits resulted in similar outcomes, there were occasions where the cell density threshold alone would not result in appropriate action, with toxin quantitation being required to confirm the overall levels of risk. As such, toxin determination is a valuable tool for assessing risk to human and animal health and should be employed when conducting full risk assessment of cyanobacterial blooms.

In this study, hydrologic manipulation, ultrasound and barley straw were all utilized by local authorities to attempt bloom control. The first intervention measure was the addition of barley straw, which was conducted prior to the first major bloom and during the onset of the first bloom in early May. The effects of these additions were unclear, given that no cyanobacteria blooms were observed after the first intervention, and with no controls in place for assessing the likelihood of blooms in untreated areas of the lake. Similarly, during the May intervention, the addition of bales was followed closely by hydrological manipulation. Cell density data did, however, indicate that the blooms reduced in mid-May after both the barley and water manipulation were enforced (Figure 5), although no statistical significance of these effects could be determined. The sluice opening conducted in June to remove water from the lake was conversely followed by a maximum cell density, but again the significance of this was clouded by the application of ultrasound waves soon after. Finally in August, the removal of debris and more sluice gate manipulation was followed by a drop in cell densities but also by another large bloom approximately four weeks later. Whilst numerous studies have utilized barley straw for the control of cyanobacteria, the effects of such treatments are variable [36,96,97].

**Figure 15:**
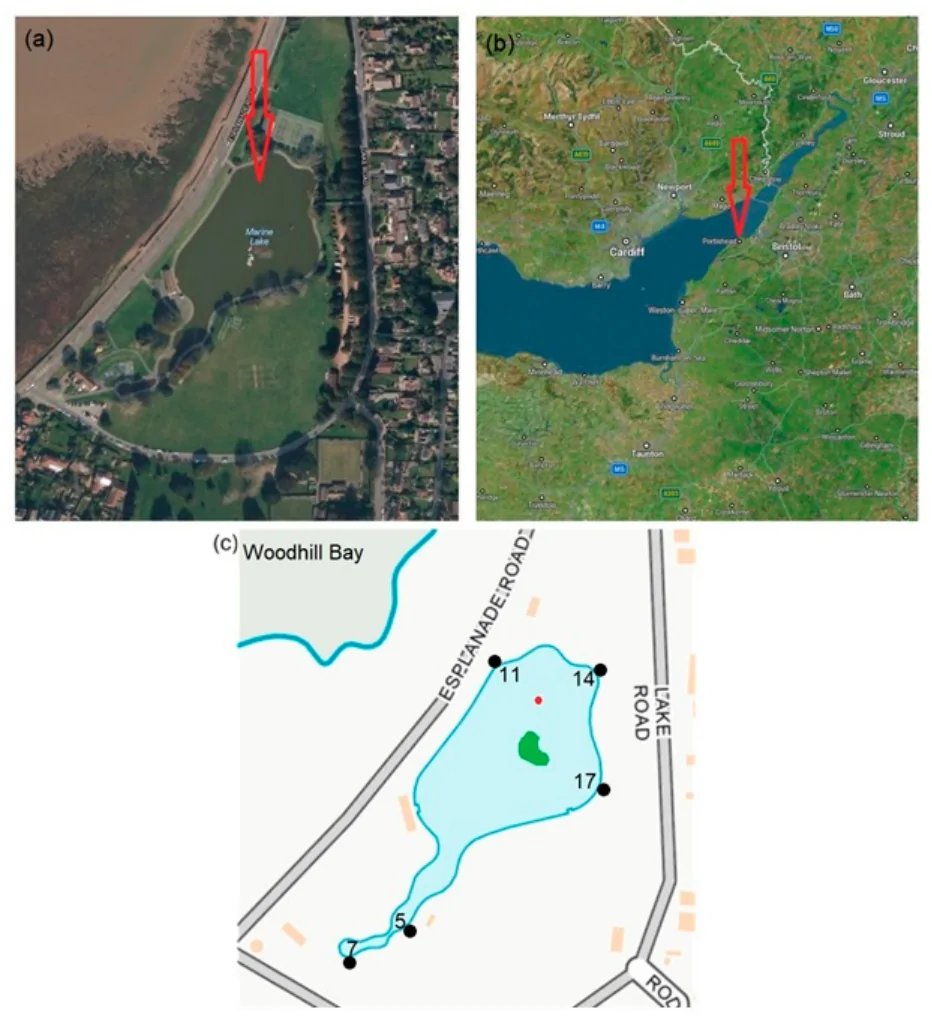
Maps showing (**a**) Portishead Marine Lake with proximity to Severn Estuary and surrounding town; (**b**) location of Portishead town in western England; (**c**) Portishead Lake showing five sampling points (5, 7, 11, 14 and 17) selected for systematic sampling throughout the study, with green shape showing mid-lake island, red dot highlighting approximate position of ultrasound device fitted during the study and black markers showing position of lakeside sampling points.

Ultrasound equipment was employed during this study and was located north of the lake centre, lying closest to Sites 14 and 11, consequently being furthest from Sites 5 and 7 at the southern end (Figure 15). The success of the technique is known to be highly variable, however, depending on ultrasound frequency, power, time and water volumes [98], but with some studies reporting over 60% of *Microcystis* population removal during treatment [99]. In terms of mean *Microcystis* spp. cell densities, implementation of the ultrasound in mid-June was followed closely by a major bloom two weeks later, and again later in the year during August and September. However, without the presence of a control area enabling the comparison of treated vs. untreated water, it was not possible to determine whether this treatment effect was significant or not. Similarly, whilst it was noted that the ultrasound device was closest to Sites 11 and 14, and that these two sites had lower median toxin concentrations than the other sites (with the exception of the enclosed children’s paddling area at Site 7), there was no way to differentiate the potential effects of ultrasound treatment in comparison to other environmental effects such as wind direction. It was also notable that high cyanobacterial cell densities and toxin concentrations continued to be quantified at both Sites 11 and 14 at various time points post-ultrasound installation (Figure 8; Tables S2 and S4).

Overall, with limited evidence for the effectiveness of the ultrasound treatment and barley straw addition, the most effective mitigation in this lake study appeared to be the removal of water through sluice gates, reducing the cyanobacterial biomass at some, but not all, treatment points. However, given the repeated occurrence of blooms subsequent to such treatment, water managers either need to be prepared to conduct hydrological intervention on a frequent basis, or should look at other measures, particularly longer-term solutions for reducing nutrient levels within the lake.

### 3.4. Human Health Impacts

Portishead Marine Lake had experienced repeated outbreaks of cyanobacterial blooms during the 2010s according to anecdotal reports, media and verbal information provided to the study team by local authority officers. Media reports described serious risks to lake visitors from exposure to toxic cyanobacteria, the loss of pet lives and well-being and significant ecological damage including fish and bird mortality and morbidity. Furthermore, significant socio-economic impacts were noted causing great concern in the local community, with the local authorities preventing access to the lake and all related recreational activities being banned [100]. Whilst the lake is not used for open-water swimming, boating activities take place and children play in the shallow end (Site 7). Routes of exposure therefore can include water ingestion through accidental swallowing, inhalation and skin contact. Whilst to the author’s knowledge, no human health issues were reported to the medical services during this study, there were still significant risks to humans as a consequence of the toxin-producing cyanobacterial blooms evidenced and the high intracellular toxin concentrations quantified.

The 20,000 cells/L cyanobacterial threshold associated with relatively low probability of health risks and used to trigger preventative action was exceeded during 15 different weeks. The GV associated with moderate probability of adverse health effects, associated with a cyanobacterial cell density of 100,000 cells/mL, was exceeded in nineteen different water samples across twelve different weeks. This implies that there were significant short-term and potential for long-term illness in humans. These risks were further increased through the identification of cyanobacterial scums along the water surface and lake edges, resulting in a 1000-fold accumulation of toxic cyanobacteria and changing cyanotoxin risk from moderate to high [20,77,101]. The breaching of this level indicates the potential for acute human poisoning and long-term illness.

Given that some cyanobacterial species are not associated with toxin production, it was also important to quantify toxin concentrations within the water samples. In relation to toxin presence through the study, total MC concentrations can be compared against the WHO provisional GV, noting that GVs are only derived for the MC-LR variant, given the insufficient data available for other MC variants and the use of uncertainty factors [21,101,102,103]. The provisional lifetime and short-term drinking-water GVs for MC-LR are 1 µg/L and 12 µg/L, respectively [102]. Subsequently, the WHO proposed a provisional recreational water GV for MC-LR of 24 µg/L based on the same NOAEL, which was based on the exposure of a child (defined as 15 kg in weight and with a default 250 mL water ingestion amount) [77]. Intracellular summed MC concentrations were found to be approximately equal to the GV in 24 different samples collected, showing significant potential human health risks from toxins present in live cellular material. Furthermore, whilst extracellular toxin levels were proportionally lower, with maximum concentrations exceeding 20 µg/L, there are potential additional health impacts from dissolved toxins in the lake water. Whilst open-water swimming is not allowed in this particular lake, exposure through ingestion, inhalation and direct water contact is still possible as a consequence of boating on the lake and children playing in the water, implying additional health risks may have originated from both intracellular and extracellular toxins.

Given that the toxin congeners detected in this study were wide-ranging, there needs to be a way to determine exposure risks based on the toxicology of complex MC mixtures. However, there is an absence of oral toxicity data for other MC analogues, so the recommendation is to apply the GV based on the assumption that all MCs have equivalent toxicity to MC-LR. Consequently, there are additional uncertainties present relating to the likely levels of toxicity in humans when complex MC mixtures are determined within cyanobacterial samples [1,77].

Overall, the data generated in this study show clear evidence for risks to human health. With the *Microcystis* spp. present containing high MC concentrations periodically, it was highly appropriate for local authorities to both conduct attempts at bloom mitigation and to prevent access to the recreational waters for the general public.

## 4. Conclusions

This study reports on the year-long assessment of cyanobacteria and cyanotoxins in a small eutrophic lake in SW England that is utilized for recreational activities such as boating and paddling. Portishead marine lake had been affected by repeated prolific cyanobacterial blooms since 2010, with annual lengthy closures of the lake to visitors and subsequent negative socioeconomic impacts. During 2016, weekly water samples were taken for cyanobacteria and cyanotoxin testing, along with environmental monitoring conducted by citizen scientists. Results revealed the prevalence of *Microcystis* species throughout the year, with several maxima in cell densities, and with intra-lake variability in biomass. Analysis of *Microcystis* cellular material evidenced high microcystin toxin concentrations within the lake, with complex toxin congener profiles and relatively low extracellular toxin content. Samples containing total MCs above the WHO recreational water guidance limit of 24 µg/L were all associated with samples containing cell densities above the current cyanobacterial action limit of 20,000 cells/mL. However, several water samples showed cell densities above the threshold, but with low toxin concentrations present, indicating that the quantitation of toxin levels was the most effective method of monitoring health risk. Toxin concentrations were found to be correlated most closely with nitrate concentrations and were unexpectedly inversely proportional to pH. There was limited evidence for any statistical relationships between toxins and environmental parameters such as wind or rainfall, although all toxic samples originated in water samples collected during periods of higher temperature. High nutrient concentrations were likely caused by high densities of seabirds and migrating wildfowl that live around and within the lake. As a consequence of the prolific blooms, local authorities prevented public access to the lake at certain times of the year. Intervention measures taken included water removal via sluice gate opening, debris removal, the use of barley straw and the installation of ultrasonic treatment part-way through the year. Whilst statistical assessment of intervention impact was not possible without effective control samples or any historical cyanobacteria and/or cyanotoxin concentration data, there was some evidence for temporary reduction in toxin concentrations following hydrological intervention, but with little indication of the success of the ultrasonic treatment or use of barley straw. It is important to stress, however, that without controls in place, the effectiveness of any mitigation measure assessment was impossible to determine. Further work should be undertaken to assess the effectiveness of these and other intervention measures, along with the ongoing long-term monitoring of cyanobacteria and toxins in the lake waters.

## 5. Methods and Materials

### 5.1. Chemicals and Reagents

Chemicals and reagents used in this study were reagent-grade as a minimum. Methanol used for cyanobacterial pellet extraction was HPLC-grade. Instrument solvents used for chromatographic mobile phase preparation were LC-MS-grade (Fisher Optima, ThermoFisher, London, UK), and all chemicals were LC-MS reagent-grade where possible; otherwise, HPLC-grade. Cyanotoxin reference standards were all obtained from Enzo Life Sciences (Enzo, Exeter, UK) and comprised the microcystin analogues (MC-RR, MC-LA, MC-LY, MC-LF, MC-LW, MC-YR, MC-WR, MC-HilR, MC-HtyR, MC-LR, [Asp3] MC-LR) and Nodularin-R (Nod-R). Enzo reference standards received as solid powders were dissolved in suitable volumes of 50% aqueous methanol to form concentrated stock solutions. A mixed stock solution was prepared by combining aliquots of each stock, with calibration solvent standards prepared resulting in a seven-point calibration range between 1.0 ng/mL to 500 ng/mL per toxin.

### 5.2. Sample Setting and Water Sampling

Portishead Marine Lake is situated in England with coordinates 51°29′27″ N latitude, 002°46′21″ W longitude (Figure 15). The lake is 16,000 m^2^ with a maximum depth of just over 1 m and situated within a grassland site, edged by woods, with a range of public amenities and overlooks the sea along the eastern shore of the Severn Estuary. The lake is filled naturally with springs running down from the surrounding land, hills and residential areas, and water can be emptied from the lake if levels rise too high, with a sluice gate that directs outfall into the sea. The lake and surrounding grounds are considered a “Formal Park and Public Garden” and is recognized as providing an extensive recreational space for the community with high importance for both locals and tourists [34]. As well as being used for recreational purposes, the lake incorporates a natural population of birds, including swans, gulls, ducks and geese, many of whom nest on the island near the lake centre. It is considered an important nesting area for visiting breeding waterfowl and also contains populations of fish.

Following an initial pre-study scoping visit when 20 different sampling points were defined, five different sites around the shore of the lake were chosen for ongoing routine sampling. Sites 11, 14 and 17 were chosen at the NW, NE and SE corners of the lake, respectively, including leeward sites where any cyanobacterial scums were likely to collect, fitting with the Environment Agency protocol of sampling the worse-case scenario. Similarly, Site 5 was taken to cover the SW corner where the lake becomes most restricted, with potential for blooms to be concentrated. An additional Site 7 was included in the shallow children’s paddling area at the far SW of the water body, which has an open but restricted connection to the main lake at Site 5 (Figure 15). The choice of these five sites was made to cover the main corners of the lake, and these were next to safe areas on land where sampling officers and citizen scientists could retrieve water samples without falling in. A maximum of five sites was possible given the practical and financial limitations on the total number of samples and subsequent analyses that could be conducted, both in the laboratory and by citizens.

Water samples were taken on a weekly basis, where possible, by the Environment Agency, between 21/1/2016 and 23/11/16. When taking samples, the time of day was recorded along with any visual notes regarding the presence of cyanobacteria in the water. Five sampling sites were visited consistently throughout the year with no spatial deviations; Sites 5, 7, 11, 14 and 17 (Figure 15), resulting in five samples taken each week. Each individual sample consisted of 50 mL water scooped from the surface of the lake into a polypropylene centrifuge tube. If cyanobacteria/algal cells were evident visually, then these cells were incorporated where possible within the water sample, along with any scum if present. Once collected, tubes were capped, sealed and sent by either same-day courier shipment or next-day Royal Mail delivery to the Cefas laboratory for processing.

### 5.3. Environmental Analysis (Weather, Temperature, etc.)

The lake was also visited by local citizen scientists on a weekly basis, who were responsible for conducting environmental monitoring of the area. The lake water was analyzed at each of the five monitoring sites for temperature and dissolved oxygen concentrations. A Fisher Scientific (Loughborough, UK) Fisherbrand portable dissolved oxygen (DO) metre (model 06-662-66) was used that had been calibrated prior to use. The metre was capable of measuring DO over a range of 0.0 to 20.0 mg/L, with a resolution of 0.1 mg/L and an accuracy of ±0.4 mg/L, with a salinity range of 0% to 39%. The unit also measured water temperature over a range of 0 °C to 60 °C, with an accuracy of ±0.8 °C [104].

In addition, an Aercus WS3084 professional wireless weather station instrument (Greenfrog Scientific, Doncaster, UK (www.greenfrogscientific.co.uk, accessed on 4 October 2026)) was used for recording meteorological conditions each week. Parameters measured were air temperature °C (mean, mean minimum, mean maximum, minimum and maximum), number of air frost days, mm rainfall (total, amount on wettest day, number of rainy days and dry days), wind speed (average speed, highest gust, date of highest gust, wind run (nm) and number of gale days), maximum and minimum air pressure in hectopascals (hPa) and total hours of sunshine per week. The instrument was installed 2.77 km from the lake on a permanent structure at a total height of 4 m above ground. Instrument specifications: temperature range −40 °C to +60 °C, with resolution 0.1 °C; measuring range of relative humidity 10% to 99%; rain volume resolution 0.3 mm; wind speed 0–160 kph; light 0–400k Lux; measurement intervals 48–60 s; air pressure range 300–1100 hPa (8.85–32.5 inHg), resolution 0.1 hPa.

### 5.4. Water Chemistry (pH, Micronutrients)

Water samples received at the laboratory were opened, and the pH was measured in each sample using calibrated pH metres. Each sample was homogenized through repeated gentle inversion before a 2.0 mL aliquot was taken for microscopy. At this stage, samples were subjected to centrifugation at 4500× *g* for 10 min, with the supernatant subsequently decanted into a second tube, leaving the remaining pellet at the bottom of the original sample collection tube. A 6 mL aliquot of the supernatant of each sample was subjected to portable nutrient testing using commercial test kits, specifically for phosphate, nitrate, nitrite and ammonia. Tests were sourced from Simplex Health ^®^ (Wellingborough, UK), provided in packs of 25 or 50 strips for each assay. Test strips were placed into water samples and left for the amount of time according to the manufacturer’s instructions.

### 5.5. Cyanobacterial Analysis

#### 5.5.1. Utermohl Microscopy

Water/algae sub-samples were fixed through addition of Lugol’s iodine solution and allowed to warm to room temperature before gentle inversion to homogenize immediately prior to analysis. Analysis was then conducted using Utermöhl’s counting technique with a high-powered inverted microscope (Olympus IX83 inverted microscope equipped with Olympoys XC70 camera, Olympus Corporation, Tokyo, Japan) and Sedewick Rafter counting cell slides [105,106]. Any recognized nuisance algae or cyanobacterial genus present were recorded. Cell counts were performed by determining the total number of organisms visible in the chamber or chamber sub-sections, facilitating the calculation of cell densities in terms of cells/mL. When cell colonies were visible upon first inspection, the fixed samples were left for several days or more and subjected to periodic shaking until colony disintegration was observed. Cell counts were then performed on separated cells to enable the more accurate estimation of cell densities [107].

#### 5.5.2. Use of IOLight Portable Microscopy

At the time of the water sampling, the IOLight portable microscope was not commercially available. Post-study assessment of the capabilities of the equipment for citizen scientists was subsequently conducted using environmental freshwater samples containing *Microcystis* spp. blooms. An IOLight compact inverted microscope was utilized for this assessment. Field of view = 1 mm × 0.75 mm; 1 micron resolution, equivalent to ×10 onjective 0.25NA [108].

### 5.6. Toxin Analysis

#### 5.6.1. Sample Processing

Both intracellular and extracellular toxin concentrations were assessed following the method of [109]. The former were analyzed through the analysis of sub-samples taken from the post-centrifuge supernatants following filtration of a minimum of 1.5 mL through a 0.2 µm syringe filter directly into an LC-MS glass autosampler vial. For any samples showing floating cells after centrifugation, a filtration method was used to collect cells, rather than centrifugation [109]. In brief, the water samples were mixed gently and then filtered through a 0.2 µm Teflon filter using a vacuum pump.

The cell pellets remaining in the 50 mL centrifuge tube after removal of supernatant were quantitatively transferred to a 2 mL Eppendorf tube after mixing with a total of approximately 1 mL of the supernatant per sample. Samples were centrifuged at 12,500× *g* for 10 min, before the supernatants were carefully removed and the remaining cell pellets placed into a freezer (<−16 °C) for a minimum of 1 h to help cell lysis. The tubes were subsequently removed from the freezer, allowed to thaw to room temperature, and 1.0 mL 80% MeOH was added to each. Each tube was vortex mixed for 60 s, before leaving for a further 30 min. Following a second vortex mix, tubes were re-centrifuged (12,500× *g*; 10 min) and the supernatants filtered through clean 0.2 µm syringe filters into glass autosampler vials for intracellular toxin analysis. For cells collected by filtration, filters were placed into clean 50 mL centrifuge tubes and stored in the freezer for a minimum of 1 h. After removing from the freezing and thawing to room temperature, 5.0 mL 80% MeOH was added to each filter, and the tubes were gently shaken before leaving in the solvent for 1 h with occasional gentle mixing to dissolve the intracellular toxins. After soaking, the solvents were filtered through a 0.2 µm syringe filter into an LC-MS autosampler vial prior to extracellular toxin analysis.

#### 5.6.2. LC-MS/MS

Water (extracellular) and pellet (intracellular) fractions were analyzed using targeted ultra-high-performance LC-MS/MS. This involved reverse-phase UHPLC for separation of the MCs and Nod-R using the validated and ISO17025-accredited method of [109]. A Waters (Manchester, UK) Xevo-TQ triple quadrupole mass spectrometer (MS/MS) coupled to a Waters Acquity UHPLC system was used for LC-MS/MS. Chromatography was conducted using a 1.7 μm, 2.1 × 50 mm Waters (Manchester, UK) Acquity UPLC BEH C18 column (P/N 186002350) together with a Waters (Manchester, UK) VanGuard BEH C18 1.7 μm, 2.1 × 5 mm guard cartridge (P/N 186003975). The column oven was set to +60 °C and water samples held at +10 °C until injection. The mobile phase flow rate was 0.6 mL/min and the sample injection volume was 5 μL. Mobile phase A was water + 0.025% formic acid, with mobile phase B acetonitrile (MeCN) + 0.025% formic acid. The chromatographic gradient was as described by [109], with a total run time of 6.5 min. Each instrumental sequence started with a series of instrumental blanks, followed by toxin calibration standards. After running sample extracts, instrumental sequences finished with a second set of calibration standards. The MS/MS source conditions for the Waters Xevo TQ were as follows: 150 °C source temperature, 600 °C desolvation temperature, 600 L/h desolvation gas flow, 0.15 mL/min collision gas flow. Capillary voltage was held at 1.0 kV. Selected reaction monitoring (SRM) transitions were built into the MS/MS method using positive mode acquisition for each toxin. Parent and daughter ions, as well as cone and collision voltages, were based on those described by [109]. Quantitation of toxins was performed against external calibration standards, calculated in terms of ng/mL of sample extracts and dilution/concentration factors used to calculate toxin concentrations within the initial water sample as received. Selected Reaction Monitoring (SRM) transitions were as summarized in Table 2. Quantitative intracellular and extracellular toxin concentrations were determined. The proportion of extracellular vs. intracellular toxin concentrations was calculated by directly comparing the two concentrations with the summed total concentration of extracellular and intracellular combined.

### 5.7. Ultrasound Treatment

The lake was fitted with an HZ Sonic (Northampton, UK) ES300 ultrasound unit containing an ultrasound 40 W power control box and a transducer. It was fixed to a float raft and anchored into a fixed position within the lake, powered by a solar cell. The unit is a titanium and stainless steel box, fitted with waterproof cables and connectors. The maximum operating range is 300 m, with the manufacturers stating it is capable of killing and controlling a range of algae types in smaller ponds and pools [110]. The ultrasonic frequency bandwidth is between 28 and 40 kHz, with 90% of the power output at those two frequencies.

## Figures and Tables

**Table 1:** Summary of physical intervention conducted in lake over the study period.

Date	Intervention Measures
14 March	60 new barley bales added across the lake.
10 May	Barley cleared from Site 5 to allow flow from Site 7. Five new barley bales added at Site 5.
11 May	Sluice from main lake opened for a few hours to allow flow out of the lake.
13 May	Sluice from main lake opened for a few hours to allow flow out of the lake.
24 May	Sluice from main lake opened for a few hours to allow flow out of the lake and drop water level
7 June	Leaf, branch and litter debris removed from lake
14 June	Sluice from main lake opened for a few hours to allow flow out of the lake and drop
16 June	Solar powered ultrasound equipment installed in centre of lake.
19 August	Unblocked Site 7 overflow and cleared debris from Site 5 during period of low water level.
23 August	Sluice gate workings inspection. Small amount of water allowed to flow through.

**Table 2:** Summary of mass spectral conditions utilized for determination of each cyanotoxin using LC-MS/MS methods.

Toxin	SRM/SIM Transitions	Cone, V	Collision Energy (eV)
MC-RR	519.9 > 134.9; 126.9	30	40; 60
Nod-R	825.5 > 135.1; 103.1	55	60; 100
MC-LA	910.1 > 135.1; 106.9	35	65; 80
[Asp3]-MC-LR	981.5 > 134.9; 106.9	75	60; 60
MC-LF	986.5 > 213.0; 135.0	35	60; 65
MC-LR	995.6 > 135.0; 127.0	60	65; 80
MC-LY	1002.5 > 135.0; 106.9	40	75; 80
MC-HilR	1009.7 > 134.9; 126.9	75	40; 60
MC-LW	1025.5 > 134.9; 126.8	35	65; 90
MC-YR	1045.6 > 135.0; 126.9	75	75; 80
MC-HtyR	1059.6 > 134.9; 106.9	75	75; 80
MC-WR	1068.6 > 134.9; 106.9	80	65; 80

## Data Availability

The original contributions presented in this study are included in the article. Further inquiries can be directed to the corresponding author.

## Supplementary Materials

The following supporting information can be downloaded at: https://www.mdpi.com/article/10.3390/toxins18100442/s1. Figure S1: Photographs of lake highlighting several features of note: (a) gulley added to reduce nutrient input into lake (post-study?); (b) drain providing water into lake at Site 14 from surrounding grounds; (c) birds and bird guano along path close to Site 5; (d) Site 7 shallow paddling pool; (e) island at lake centre; (f) Site 17 showing accumulation of rubbish and debris within lake water; (g) close up of ultrasonication device, north of island; Table S1: Summary of meteorological data throughout study period showing temperature (°C), rainfall (mm), wind (kts), air pressure (hPa) and sunshine (h); Table S2: Summary of water samples showing sampling notes recorded at time of sampling, and the Microcystis species cell densities enumerated following light microscopy analysis; Table S3: Summary of water chemistry results determined from Portishead Lake, showing pH, dissolved oxygen concentrations (DO; mg/L) and water temperature (°C), together with phosphate, nitrate and nitrite concentrations (mg/L); Table S4: Intracellular microcystin concentrations (µg/L) quantified in water samples from Portishead Lake; Table S5: Summary of extracellular microcystin concentrations quantified in lake water samples; Table S6: Parsimonious linear mixed-effect model output; Figure S2: Parsimonious linear mixed-effect model residual plot; Figure S3: Site effect on log intracellular MC concentrations from linear mixed-effect model output; Figure S4: Random Forest variable importance plot; Figure S5: Parsimonious linear model output and ANOVA on effect of site on MC concentrations; Figure S6: Tukey’s post hoc analysis on effect of individual site pairs on MC concentrations.
