# Supplementary material for: Risks to Recreational Water Users from Microcystis spp. and Microcystins in Portishead Lake (SW England) as Determined by LC-MS/MS and Citizen Science Monitoring
Source: Toxins (Basel). 2026 Oct 13;18(10):442. doi: 10.3390/toxins18100442 (PMC13709399; doi:10.3390/toxins18100442)
Supplement: Supplementary file 1 [file toxins-18-00442-s001.zip › toxins-4459023-supplementary.pdf]

Supplementary Materials: Risks to Recreational Water Users from *Microcystis* spp. and Microcystins in Portishead Lake (SW England) as Determined by LC-MS/MS and Citizen Science Monitoring

Andrew D. Turner, Karl J. Dean, Monika Dhanji-Rapkova, Alison O'Neill, Benjamin H. Maskrey, Patrycja Stubbs, David M. Hartnell, Shamina Page, Naomi Baker, Nadine Morrell, John M. Turner, Joan Turner, Florence R. I. Turner, Stephen D. Gregory, Adam M. Lewis and Katy Lewis

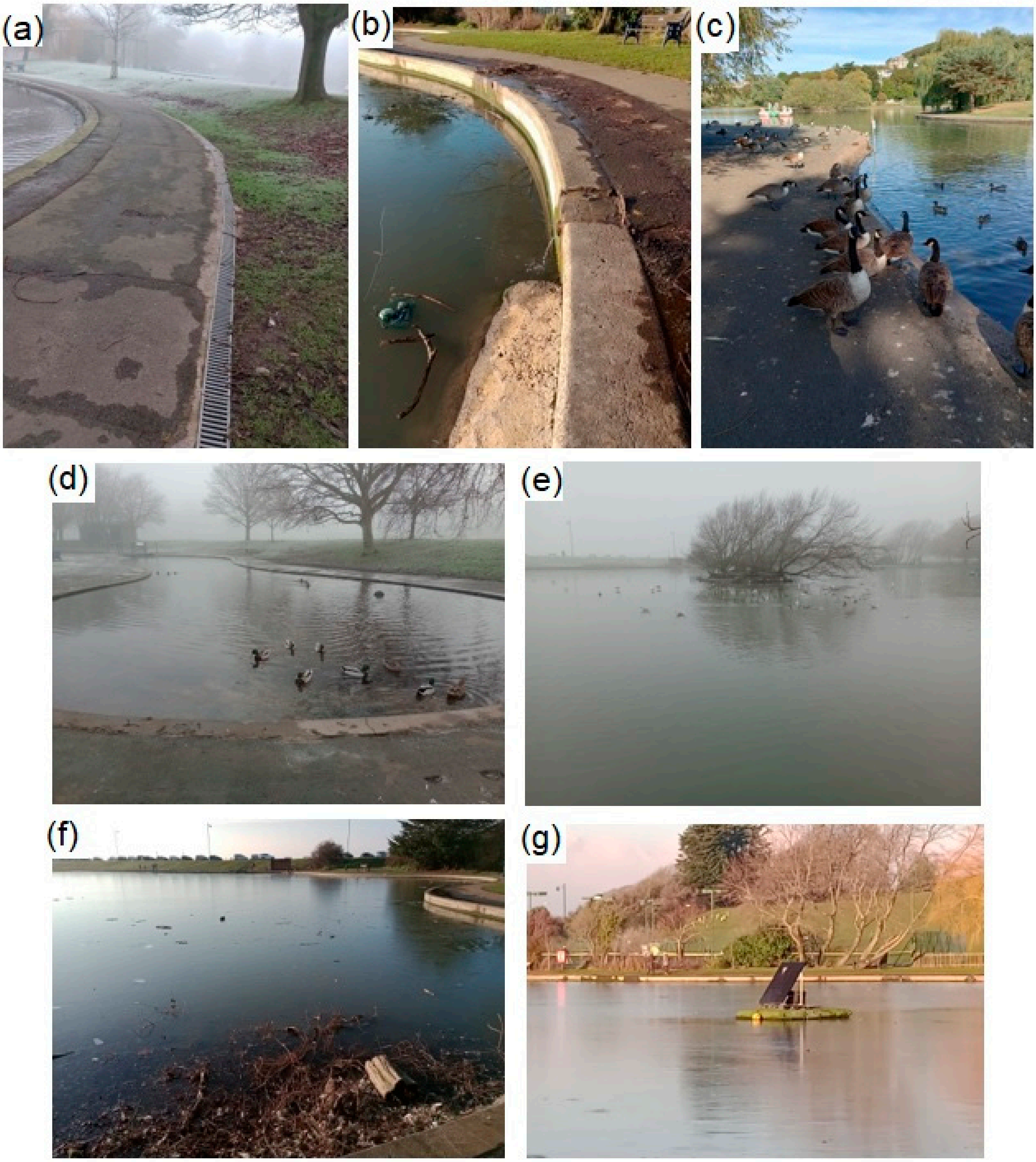

Figure S1. Photographs of lake highlighting several features of note: (a) gulley added to reduce nutrient input into lake (post-study?); (b) drain providing water into lake at Site 14 from surrounding grounds; (c) birds and bird guano along path close to Site 5; (d) Site 7 shallow paddling pool; (e) island at lake center; (f) Site 17 showing accumulation of rubbish and debris within lake water; (g) close-up of ultrasonication device, north of island.

Table S1. Summary of meteorological data throughout study period showing temperature (°C), rainfall (mm), wind (kts), air pressure (hPa) and sunshine (hr).

	Temperature (°C)						Rainfall (mm)				Wind (kts)				Pressure (hPa)		Sunshine
Date	Mean	Mean Min	Mean Max	Min	Max	Air frosts	Total amount	Amount on wet-test day	Rain days	Dry days	Av speed	High-est gust	Wind run (nm)	Gale days	Max	Min	Total hours
23/01/2016	2.5	0.6	6.5	-2.8	8.4	5	0	0	0	12	1.3	4.1	142	0	1029.7	1018.2	18.2
05/02/2016	3.4	2.3	9.2	-0.2	13.3	2	0	0	0	9	1.8	6.5	203	0	1027.4	1014.3	21.3
14/02/2016	5.4	3.4	12.3	0.6	14.9	1	2.3	2.3	1	11	3.5	12.1	298	0	1021.3	1004.3	22.4
26/02/2016	6.1	3.8	11.4	1.9	13.4	0	0	0	0	4	1.4	7.8	245	0	1022.8	1016.5	14.6
02/03/2016	9.5	6.5	13.8	4.3	17.2	0	0	0	0	12	2.3	9.4	342	0	1020.1	1017.2	28.3
15/03/2016	10.3	5.9	12.9	6.2	15.6	0	5.8	3.9	2	7	3.1	15.6	387	0	1023.8	1004.6	19.9
24/03/2016	9.8	6.5	14.1	5.4	18.1	0	3.8	2.3	2	6	2.9	10.1	241	0	1024.6	1002.6	21.2
02/04/2016	10.9	7.2	15.7	6.3	18.3	0	6.5	4	3	12	3	14.3	365	0	1023.7	1005.6	18.3
17/04/2016	11.6	7.4	16.8	5.6	18.4	0	0	0	0	3	3.9	16.5	211.6	0	1026.3	1016.7	21.7
21/04/2016	9.2	5.4	14.6	3.4	16.9	0	8.4	5.1	5	3	3.1	23.1	555.1	0	1022.2	1005.6	38.5
29/04/2016	10.6	6.4	15.4	3.7	20.3	0	2.7	2.1	2	4	3.9	17.9	574.8	0	1024.1	1005.9	41.5
05/05/2016	16.0	12.1	21.8	6.8	27.9	0	40.8	10.8	5	2	1.9	10.5	299.7	0	1018.9	998.9	32.5
12/05/2016	14.0	9.8	19.4	7.2	22.4	0	9	6.9	4	3	3.4	15.2	578.6	0	1019.8	999.4	53.6
19/05/2016	13.6	10.1	18.1	7.8	20.7	0	18.9	13.5	4	3	3.3	15.2	571.7	0	1019	1002	31.6
26/05/2016	16.1	12.1	21.6	10.4	23.5	0	5.1	3	2	5	2.5	12.6	395.1	0	1020	1009.1	46.6
02/06/2016	17.6	13.2	24.1	9.3	27.9	0	0	0	0	6	2.5	14.6	428.4	0	1021	1012	51.5
09/06/2016	17.2	14.3	22.9	11.8	27.0	0	14.4	5.1	6	1	2.8	16.5	486.5	0	1019.1	991.6	20
16/06/2016	16.5	13.7	21.0	11.7	24.2	0	22.5	4.1	4	3	1.1	6.0	575.9	0	1019.8	994.6	38.0
24/06/2016	16.5	13.7	21.0	11.7	24.2	0	22.5	4.1	4	3	1.1	6.0	575.9	0	1019.8	994.6	34.2
01/07/2016	16.5	13.7	21.0	11.7	24.2	0	22.5	4.1	4	3	1.1	6.0	575.9	0	1019.8	994.6	32.6
07/07/2016	17.7	14.9	22.6	12.1	24.6	0	12.3	7.5	3	4	3.9	19.8	685.8	0	1018.7	1001.2	30
14/07/2016	20.0	15.8	25.5	12.9	35.0	0	0.6	0.6	1	6	3.1	15.9	533	0	1023.4	1002	50.1
21/07/2016	19.3	16.2	24.1	15.4	29.3	0	4.2	3	3	4	3.1	14.6	534.4	0	1018.4	1010.9	44

28/07/2016	18.0	14.9	22.5	12.4	27.7	0	38.7	29.4	4	2	3.4	15.2	462.9	0	1016.5	1004.9	30
03/08/2016	18.5	15.3	22.6	12.6	24.5	0	3.6	3.6	1	6	4.2	17.1	736.1	0	1024	1003.3	51.5
10/08/2016	18.8	14.1	24.8	12.6	28.6	0	0.9	0.9	1	7	2.4	13.8	488.6	0	1023	1006.7	64.5
18/08/2016	19.2	15.7	23.8	14.2	31.0	0	23.4	12.6	5	2	3.4	19.8	596.3	0	1020.3	998.3	34.5
25/08/2016	18.9	14.8	24.5	13.6	30.7	0	7.8	6	3	4	2.1	13.2	369.8	0	1021.5	1007.1	50
01/09/2016	18.5	15.6	22.1	14.0	26.1	0	33	24.9	5	2	3.1	17.1	516.8	0	1020.5	991.5	29.1
08/09/2016	18.9	15.1	23.3	12.4	28.5	0	na	na	na	na	2.9	15.2	504.9	0	1013.9	1003.9	41.1
18/09/2016	16.5	13.8	21.3	13.2	23.9	0	3.6	3.6	1	4	1.2	9.3	112	0	1019.4	1011.5	15
22/09/2016	16.2	13.4	19.5	11.8	20.8	0	18.9	12.3	5	2	3.6	14.6	595.5	0	1021.5	1003.2	39.9
29/09/2016	14.3	10.5	20.0	8.1	22.8	0	6.9	2.7	3	4	2.7	16.5	455	0	1024.7	1000.4	55.1
06/10/2016	12.1	8.9	17.0	6.5	19.2	0	1.2	1.2	1	6	2	11.3	315.8	0	1026	1014.2	31.5
13/10/2016	12.1	9.6	16.4	8.0	19.2	0	21.3	8.1	4	4	2.6	13.2	500.7	0	1020.4	999.2	42.7
20/10/2016	10.8	8.6	14.2	6.7	18.5	0	11.4	7.2	3	4	2.1	13.2	329.2	0	1027	1005	24.2
27/10/2016	12.2	10.1	15.1	6.0	16.8	0	0.6	0.3	2	5	1.8	13.8	311	0	1031.4	1019.5	22
03/11/2016	8.3	5.5	12.3	3.3	14.6	0	45	22.5	4	4	2.3	11.3	442.8	0	1020.7	900.7	39.7
10/11/2016	11.2	8.7	14.7	5.4	20.1	0	10.8	6.9	3	4	3.6	14.6	557.5	0	1027.3	1003.1	31
17/11/2016	7.1	5.4	9.4	4.0	12.4	0	138.3	69.6	5	1	4	21.8	639.6	0	1017.7	973.1	22.6
24/11/2016	6.1	3.9	9.9	-0.7	12.6	1	39	33	3	4	2.8	19.2	496.8	0	1032.1	980	29.6

na = no analysis due to temporarily malfunctioning rain gauge.

Table S2. Summary of water samples showing sampling notes recorded at time of sampling, and the *Microcystis* species cell densities enumerated following light microscopy analysis.

Date	Site	Sampling notes	Cells/mL
21/01/2016	Site 5	Blooms under ice	60000
	Site 7	Blooms under ice	120000
	Site 11		1600
	Site 14		2000
	Site 17		
28/01/2016	Site 5	Less algal matter	
	Site 7	Less algal matter	
	Site 11	Less algal matter	
	Site 14	Less algal matter	
	Site 17	Less algal matter	
03/02/2016	Site 5	No blooms visible	
	Site 7	No blooms visible	
	Site 11	No blooms visible	
	Site 14	No blooms visible	
	Site 17	No blooms visible	
11/02/2016	Site 5		
	Site 7		
	Site 11		
	Site 14		
	Site 17		
18/02/2016	Site 5		
	Site 7		
	Site 11		
	Site 14	Slight bloom	
	Site 17		
25/02/2016	Site 5	Slight bloom	
	Site 7		
	Site 11		
	Site 14		
	Site 17	Significant bloom	2000
04/03/2016	Site 5	No visible bloom	
	Site 7	No visible bloom	
	Site 11	No visible bloom	
	Site 14	No visible bloom	
	Site 17	No visible bloom	
08/03/2016	Site 5	No visible bloom	
	Site 7	No visible bloom	

	Site 11	No visible bloom	
	Site 14	No visible bloom	
	Site 17	No visible bloom	
17/03/2016	Site 5b	Bloom and barley	
	Site 7	No visible bloom	
	Site 11	Barley	
	Site 14	No visible bloom	
	Site 17	No visible bloom	
24/03/2016	Site 5	No visible bloom	
	Site 7	No visible bloom	
	Site 11	No visible bloom	
	Site 14	No visible bloom	
	Site 17	No visible bloom	
30/03/2016	Site 5	No visible bloom	
	Site 7	No visible bloom	
	Site 11	No visible bloom	
	Site 14	No visible bloom	
	Site 17	No visible bloom	
07/04/2016	Site 5	No visible bloom	
	Site 7	No visible bloom	
	Site 11	No visible bloom	
	Site 14	No visible bloom; froth present	
	Site 17	No visible bloom; froth present	
13/04/2016	Site 5	No visible bloom	
	Site 7	No visible bloom	
	Site 11	No visible bloom	
	Site 14	No visible bloom	
	Site 17	Slight bloom	
21/4/2016	Site 5	Slight bloom	41000
	Site 7	No visible bloom	
	Site 11	No visible bloom	
	Site 14	No visible bloom	
	Site 17	No visible bloom	
27/04/2016	Site 5	No visible bloom	
	Site 7	No visible bloom	
	Site 11	No visible bloom	
	Site 14	Slight bloom	38000
	Site 17	Slight bloom	
05/05/2016	Site 5	Very obvious bloom	450000
	Site 7		
	Site 11		
	Site 14		
	Site 17		

12/05/2016	Site 5	Very obvious; sulfurous smell	1480000
	Site 7		
	Site 11		
	Site 14		
	Site 17		
18/05/2016	Site 5	Bloom lessened	
	Site 7		
	Site 11		
	Site 14		74000
	Site 17		
25/05/2016	Site 5		250000
	Site 7	Bloom evidence	
	Site 11		
	Site 14	Bloom evidence	
	Site 17	Bloom evidence	
01/06/2016	Site 5	Very small bloom	65000
	Site 7		
	Site 11		
	Site 14		
	Site 17		
08/06/2016	Site 5	Water green all over lake	2220000
	Site 7		1800000
	Site 11		840000
	Site 14		
	Site 17		
16/06/2016	Site 5	No blooms present	132000
	Site 7		560000
	Site 11		
	Site 14		
	Site 17		
22/06/2016	Site 5		200000
	Site 7		
	Site 11	Small bloom	2800000
	Site 14		
	Site 17		
29/06/2016	Site 5	No blooms present	
	Site 7		
	Site 11		
	Site 14		
	Site 17		
04/07/2016	Site 5		
	Site 7		

	Site 11		
	Site 14		
	Site 17		
12/07/2016	Site 5	No blooms present	
	Site 7		
	Site 11		
	Site 14		
	Site 17		
21/07/2016	Site 5	No blooms present	
	Site 7		
	Site 11		
	Site 14		
	Site 17		
27/07/2016	Site 5		
	Site 7	Very shallow water	
	Site 11		
	Site 14	Bloom at 14 clear	
	Site 17	Small bloom at 17	
03/08/2016	Site 5		
	Site 7		
	Site 11		
	Site 14		
	Site 17		
10/08/2016	Site 5		
	Site 7	Reduced bloom at 7; shallow	
	Site 11		
	Site 14	Slight bloom	
	Site 17	Increasing bloom at 17	
18/08/2016	Site 5		
	Site 7		
	Site 11		
	Site 14		
	Site 17		
25/08/2016	Site 5	Bloom clumps around most of the lake; significant bloom at Site 5	1200000
	Site 7		
	Site 11		
	Site 14		
	Site 17		
01/09/2016	Site 5	Clumpy bloom around 11–14	
	Site 7		
	Site 11		860000
	Site 14		3500

	Site 17		
08/09/2016	Site 5	Dispersed scum around most of the lake; windy, so not clumped	
	Site 7		
	Site 11		
	Site 14		
	Site 17		
15/09/2016	Site 5	Clumps of algae bloom around whole lake	5600000
	Site 7		
	Site 11		65000
	Site 14		423000
	Site 17		660000
22/09/2016	Site 5	Clumpy bloom at 5 & 17 only	2100000
	Site 7		
	Site 11		
	Site 14		
	Site 17		90000
29/09/2016	Site 5	No blooms	
	Site 7		
	Site 11		
	Site 14		
	Site 17		
06/10/2016	Site 5		
	Site 7		
	Site 11		
	Site 14		
	Site 17		
12/10/2016	Site 5	Clumpy bloom at Site 5 only	
	Site 7		
	Site 11		
	Site 14		
	Site 17		
20/10/2016	Site 5	Bloom	120000
	Site 7		
	Site 11		
	Site 14		
	Site 17	Bloom	180000
03/11/2016	Site 5	No bloom (1st time in weeks)	
	Site 7		
	Site 11		
	Site 14	Small clumps	
	Site 17	Small clumps	

09/11/2016	Site 5	No blooms present
	Site 7	
	Site 11	
	Site 14	
	Site 17	
17/11/2016	Site 5	No blooms
	Site 7	
	Site 11	
	Site 14	
	Site 17	
23/11/2016	Site 5	
	Site 7	
	Site 11	
	Site 14	
	Site 17	
01/12/2016	Site 5	Ice
	Site 7	Ice
	Site 11	Ice
	Site 14	Ice
	Site 17	

Table S3. Summary of water chemistry results determined from Portishead Lake, showing pH, dissolved oxygen concentrations (DO; mg/L) and water temperature (°C), together with phosphate, nitrate, and nitrite concentrations (mg/L).

Collected	Site	pH	DO	Water Temp	Phosphate	Nitrate	Nitrite	Ammonia
21/01/2016	Site 5	7.43	14.2	0	25	0.0	0.0	0.5
21/01/2016	Site 7	7.89	9.7	0	10	10.0	0.0	0.5
21/01/2016	Site 11	8.01	12.3	0	0	0.0	0.0	3
21/01/2016	Site 14	7.94	13.1	0	10	0.0	0.0	3
21/01/2016	Site 17	8.21	14.0	0	0	0.0	0.0	6
28/01/2016	Site 5	7.52	11.2	5	10	0.0	0.0	0.5
28/01/2016	Site 7	7.55	13.2	5	10	10.0	0.0	0.5
28/01/2016	Site 11	8.17	12.0	5	0	0.0	0.0	0.5
28/01/2016	Site 14	8.14	11.1	5	10	0.0	0.0	3
28/01/2016	Site 17	8.1	11.2	5	10	0.0	0.0	6
03/02/2016	Site 5	7.46	12.1	5	0	0.0	0.0	0.5
03/02/2016	Site 7	7.84	11.0	5	0	0.0	0.0	0.5
03/02/2016	Site 11	8.02	10.7	5	10	0.0	0.0	3
03/02/2016	Site 14	8.16	9.8	5	10	0.0	0.0	3
03/02/2016	Site 17	8.09	11.3	5	10	0.0	0.0	6
11/02/2016	Site 5	7.88	11.4	5	25	25.0	0.0	0.5
11/02/2016	Site 7	7.98	11.9	5	10	25.0	0.5	3
11/02/2016	Site 11	8.3	10.4	5	10	0.0	0.0	6
11/02/2016	Site 14	8.35	9.9	5	25	25.0	0.0	6
11/02/2016	Site 17	8.29	10.8	5	25	5.0	0.0	6
18/02/2016	Site 5	7.786	10.9	3.6	25	25.0	0.0	0
18/02/2016	Site 7	7.7	8.7	3.6	25	50.0	10.0	6
18/02/2016	Site 11	8.21	11.2	3.6	10	10.0	0.0	6
18/02/2016	Site 14	8.29	10.3	3.6	10	0.0	0.0	6
18/02/2016	Site 17	8.29	11.2	3.6	10	5.0	0.0	6
25/02/2016	Site 5	7.74	10.2	5.2	25	25.0	0.0	0.5
25/02/2016	Site 7	7.7	9.8	5.2	10	25.0	0.5	6
25/02/2016	Site 11	8.41	9.4	5.2	10	0.0	0.0	6
25/02/2016	Site 14	8.45	11.2	5.2	10	10.0	0.0	6
25/02/2016	Site 17	7.84	8.9	5.2	10	25.0	0.5	6
04/03/2016	Site 5	7.77	9.5	6.3	10	10.0	0.0	0.5
04/03/2016	Site 7	7.71	9.4	6.3	10	10.0	0.0	1
04/03/2016	Site 11	8.34	10.3	6.3	10	0.0	0.0	6
04/03/2016	Site 14	8.35	10.0	6.3	10	0.0	0.0	6
04/03/2016	Site 17	7.99	8.7	6.3	25	0.0	0.0	3
08/03/2016	Site 5	7.78	9.8	6.8	25	0.0	0.0	0.5
08/03/2016	Site 7	7.5	10.3	6.8	10	10.0	0.5	6
08/03/2016	Site 11	8.24	10.7	6.8	10	10.0	0.0	6
08/03/2016	Site 14	8.34	9.1	6.8	10	0.0	0.0	6
08/03/2016	Site 17	8.35	9.8	6.8	10	25.0	0.0	3

17/03/2016	Site 5b	7.73	10.1	11.8	10	0.0	0.0	0.5
17/03/2016	Site 7	7.53	10.0	11.8	10	25.0	0.0	6.0
17/03/2016	Site 11	8.45	10.6	11.8	10	0.0	0.0	6.0
17/03/2016	Site 14	8.48	11.2	11.8	10	0.0	0.0	6.0
17/03/2016	Site 17	8.42	9.4	11.8	10	0.5	0.0	3.0
24/03/2016	Site 5	7.69	9.3	8.9	10.0	0.0	0.0	0.5
24/03/2016	Site 7	7.74	10.3	9.6	25.0	25.0	0.0	6.0
24/03/2016	Site 11	8.07	9.9	9.5	10.0	10.0	0.0	6.0
24/03/2016	Site 14	8.11	11.5	8.9	10.0	0.0	0.0	3.0
24/03/2016	Site 17	8.11	10.4	9.2	10.0	0.5	0.0	3.0
30/03/2016	Site 5	7.6	8.7	6.5	25.0	0.0	0.0	0.5
30/03/2016	Site 7	7.4	9.9	6.5	10.0	0.0	0.5	6.0
30/03/2016	Site 11	7.98	10.2	9.7	0.0	10.0	0.0	6.0
30/03/2016	Site 14	8.03	10.6	9.5	10.0	0.0	0.0	6.0
30/03/2016	Site 17	7.94	8.9	9.6	10.0	10.0	0.0	6.0
07/04/2016	Site 5	7.88	9.4	9.2	10.0	10.0	0.0	0.5
07/04/2016	Site 7	7.95	10.4	7.4	10.0	10.0	0.5	6.0
07/04/2016	Site 11	8.21	9.8	9.2	10.0	1.0	0.0	6.0
07/04/2016	Site 14	8.22	9.8	9.3	10.0	0.0	0.0	6.0
07/04/2016	Site 17	8.06	9.6	9.2	0.0	10.0	0.0	6.0
13/04/2016	Site 5	7.8	8.4	11.2	25.0	10.0	0.0	0.5
13/04/2016	Site 7	7.54	8.8	10.8	10.0	10.0	0.5	6.0
13/04/2016	Site 11	8.11	8.6	11.7	10.0	0.0	0.0	6.0
13/04/2016	Site 14	8.14	8.7	11.7	10.0	0.0	0.0	6.0
13/04/2016	Site 17	8.13	9.0	11.7	10.0	10.0	0.0	3.0
21/04/2016	Site 5	7.37	9.9	11.1	0.0	25.0	0.5	0.5
21/04/2016	Site 7	7.3	12.2	9.1	10.0	10.0	0.5	6.0
21/04/2016	Site 11	8.04	9.7	12.6	10.0	10.0	0.0	6.0
21/04/2016	Site 14	8.05	9.5	12.5	10.0	0.5	0.0	3.0
21/04/2016	Site 17	8.08	9.8	12.1	0.0	0.0	0.0	6.0
27/04/2016	Site 5	7.66	10.7	8.5	10.0	10.0	0.0	0.5
27/04/2016	Site 7	7.83	11.4	7.4	0.0	10.0	0.5	6.0
27/04/2016	Site 11	8.26	10.5	8.9	10.0	0.0	0.0	6.0
27/04/2016	Site 14	8.05	9.4	8.7	25.0	0.5	0.0	6.0
27/04/2016	Site 17	7.98	10.0	8.7	10.0	0.0	0.0	3.0
05/05/2016	Site 5	7.11	7.2	12.7	25.0	0.0	0.0	1.0
05/05/2016	Site 7	7.45	9.9	12.0	10.0	10.0	0.0	6.0
05/05/2016	Site 11	8.19	10.3	14.0	10.0	10.0	0.0	6.0
05/05/2016	Site 14	8.12	8.2	13.8	10.0	0.5	0.0	3.0
05/05/2016	Site 17	7.92	8.0	14.0	10.0	0.0	0.0	3.0
12/05/2016	Site 5	6.75	9.8	15.2	25.0	10.0	0.0	6.0
12/05/2016	Site 7	7.79	8.5	14.1	10.0	50.0	1.0	6.0
12/05/2016	Site 11	7.91	5.2	16.5	10.0	50.0	0.0	6.0

12/05/2016	Site 14	7.88	8.8	16.6	10.0	0.5	0.0	3.0
12/05/2016	Site 17	7.97	7.1	16.4	10.0	0.5	0.0	6.0
18/05/2016	Site 5	8.01	9.1	14.4	10.0	25.0	1.0	0.5
18/05/2016	Site 7	8.14	10.4	12.7	10.0	100.0	1.0	6.0
18/05/2016	Site 11	8.13	10.9	15.2	10.0	50.0	0.0	6.0
18/05/2016	Site 14	8.08	10	15.3	10.0	0.0	0.0	6.0
18/05/2016	Site 17	8.03	10.1	15.0	25.0	0.5	0.0	6.0
25/05/2016	Site 5	7.95	10.6	12.4	10.0	10.0	0.0	3.0
25/05/2016	Site 7	8.01	10.4	13.7	25.0	50.0	0.0	6.0
25/05/2016	Site 11	7.86	10.5	15.7	10.0	10.0	0.0	6.0
25/05/2016	Site 14	9.03	9.6	15.8	10.0	0.0	0.0	6.0
25/05/2016	Site 17	9.06	9.7	15.4	25.0	0.0	0.0	3.0
01/06/2016	Site 5	7.42	9.8	13.0	10.0	0.0	0.0	3.0
01/06/2016	Site 7	8.14	10.1	14.2	10.0	25.0	0.0	6.0
01/06/2016	Site 11	8.05	9.9	15.7	10.0	0.0	0.0	6.0
01/06/2016	Site 14	8.03	9.4	15.7	10.0	0.0	0.0	3.0
01/06/2016	Site 17	8.04	9.4	15.4	50.0	0.5	0.0	6.0
08/06/2016	Site 5	8.24	10.4	20.1	25.0	0.0	0.0	0.5
08/06/2016	Site 7	8.17	9.7	16.5	10.0	25.0	0.0	6.0
08/06/2016	Site 11	8.3	9.0	22.0	10.0	0.0	0.0	3.0
08/06/2016	Site 14	8.25	9.1	22.0	10.0	0.0	0.0	6.0
08/06/2016	Site 17	8	9.3	21.6	0.0	0.0	0.0	3.0
16/06/2016	Site 5	7.77	8.9	17.6	10.0	50.0	0.0	6.0
16/06/2016	Site 7	8.33	10	15.4	10.0	50.0	0.0	3.0
16/06/2016	Site 11	8.29	9.3	18.4	10.0	10.0	0.0	3.0
16/06/2016	Site 14	8.15	8.9	18.3	10.0	10.0	0.0	3.0
16/06/2016	Site 17	8.22	8.7	18.2	10.0	0.0	0.0	0.5
22/06/2016	Site 5	7.9	na	na	10.0	10.0	0.0	0.5
22/06/2016	Site 7	7.96	na	na	10.0	50.0	0.0	0.5
22/06/2016	Site 11	7.42	na	na	10.0	50.0	0.0	6.0
22/06/2016	Site 14	7.93	na	na	10.0	10.0	0.0	6.0
22/06/2016	Site 17	7.96	na	na	10.0	0.0	0.0	3.0
29/06/2016	Site 5	7.47	na	na	10.0	10.0	0.0	3.0
29/06/2016	Site 7	8.11	na	na	10.0	10.0	0.0	3.0
29/06/2016	Site 11	7.96	na	na	10.0	10.0	0.0	3.0
29/06/2016	Site 14	7.92	na	na	10.0	10.0	0.0	3.0
29/06/2016	Site 17	8.03	na	na	10.0	0.0	0.0	6.0
04/07/2016	Site 5	8.54	9.4	17.6	0.0	10.0	0.0	0.5
04/07/2016	Site 7	8.71	12.1	16.1	0.0	50.0	0.0	6.0
04/07/2016	Site 11	8.27	7.6	19.4	0.0	0.0	0.0	0.5
04/07/2016	Site 14	8.21	7.7	19.2	0.0	10.0	0.0	3.0
04/07/2016	Site 17	8.26	7.3	19.2	0.0	10.0	0.0	3.0
12/07/2016	Site 5	8.55	9.7	15.6	10.0	50.0	0.0	6.0

12/07/2016	Site 7	7.67	9.8	15.0	10.0	50.0	0.0	0.5
12/07/2016	Site 11	8.6	9.5	16.8	10.0	0.0	0.0	0.5
12/07/2016	Site 14	8.67	8.5	16.6	10.0	10.0	0.0	0.5
12/07/2016	Site 17	8.46	9.0	16.9	10.0	0.0	0.0	3.0
21/07/2016	Site 5	7.52	9.4	19.6	10.0	10.0	0.0	6.0
21/07/2016	Site 7	8.16	9.6	16.8	10.0	50.0	0.0	3.0
21/07/2016	Site 11	8.09	9.1	20.6	10.0	0.0	0.0	3.0
21/07/2016	Site 14	8.04	9.4	20.4	10.0	10.0	0.0	6.0
21/07/2016	Site 17	7.64	9.5	20.6	10.0	10.0	0.0	3.0
27/07/2016	Site 5	8.09	6.0	17.0	10.0	10.0	0.0	6.0
27/07/2016	Site 7	8.33	10.2	16.0	10.0	50.0	0.0	3.0
27/07/2016	Site 11	8.21	5.8	19.5	10.0	0.0	0.0	3.0
27/07/2016	Site 14	8.25	7.5	19.7	10.0	0.0	0.0	3.0
27/07/2016	Site 17	8.04	8.5	19.7	10.0	10.0	0.0	0.5
03/08/2016	Site 5	8.03	9.4	17.2	10.0	25.0	0.0	0.5
03/08/2016	Site 7	7.96	5.0	18.5	10.0	0.0	0.0	3.0
03/08/2016	Site 11	7.94	9.5	18.7	10.0	0.0	0.0	6.0
03/08/2016	Site 14	7.54	6.5	18.7	10.0	0.0	0.0	0.5
03/08/2016	Site 17	7.96	5.6	18.2	10.0	0.0	0.0	0.5
10/08/2016	Site 5	8.24	5.6	14.4	10.0	50.0	0.0	6.0
10/08/2016	Site 7	7.79	11.9	15.4	10.0	10.0	0.0	0.5
10/08/2016	Site 11	8.28	6.8	16.7	10.0	0.0	0.0	0.5
10/08/2016	Site 14	8.22	7.5	16.0	10.0	0.0	0.0	3.0
10/08/2016	Site 17	8.33	7.3	15.7	10.0	0.0	0.0	3.0
18/08/2016	Site 5	7.77	6.6	20.5	10.0	25.0	0.0	3.0
18/08/2016	Site 7	7.63	9.4	17.4	10.0	0.0	0.0	3.0
18/08/2016	Site 11	8.29	7.0	21.9	10.0	0.0	0.0	3.0
18/08/2016	Site 14	8.3	6.8	21.8	10.0	0.0	0.0	3.0
18/08/2016	Site 17	8.05	5.4	21.8	10.0	0.0	0.0	3.0
25/08/2016	Site 5	7.1	4.4	17.7	0.0	100.0	0.0	6.0
25/08/2016	Site 7	8.25	4.3	17.2	10.0	25.0	0.0	3.0
25/08/2016	Site 11	7.98	5.6	20.9	0.0	0.0	0.0	6.0
25/08/2016	Site 14	8.3	4.7	21.2	0.0	0.0	0.0	3.0
25/08/2016	Site 17	8.06	5.8	20.5	10.0	0.0	0.0	0.5
01/09/2016	Site 5	8.07	5.4	16.6	10.0	100.0	0.0	0.5
01/09/2016	Site 7	8.22	3.5	14.7	10.0	100.0	0.0	3.0
01/09/2016	Site 11	7.25	9.6	17.6	0.0	100.0	0.0	6.0
01/09/2016	Site 14	7.57	4.7	17.6	0.0	100.0	0.0	6.0
01/09/2016	Site 17	8.25	5.0	17.9	10.0	25.0	0.0	6.0
08/09/2016	Site 5	8.1	3.9	18.7	10.0	10.0	0.0	3.0
08/09/2016	Site 7	8.16	4.2	16.2	10.0	10.0	0.0	6.0
08/09/2016	Site 11	8.29	4.3	19.2	10.0	10.0	0.0	6.0
08/09/2016	Site 14	8.23	4.4	18.9	10.0	0.0	0.0	6.0

08/09/2016	Site 17	8.21	5.7	19.1	10.0	0.0	0.0	6.0
15/09/2016	Site 5	6.95	3.8	17.0	10.0	100.0	0.0	6.0
15/09/2016	Site 7	7.94	4.6	15.4	10.0	100.0	0.0	6.0
15/09/2016	Site 11	7.29	5.5	18.6	10.0	100.0	0.0	6.0
15/09/2016	Site 14	7.1	7.9	18.6	10.0	100.0	0.0	6.0
15/09/2016	Site 17	7.2	4.2	19.0	10.0	100.0	0.0	6.0
22/09/2016	Site 5	6.88	5.9	15.4	10.0	100.0	0.0	6.0
22/09/2016	Site 7	7.91	7.9	15.2	10.0	25.0	0.0	6.0
22/09/2016	Site 11	8.06	5.7	16.0	10.0	10.0	0.0	3.0
22/09/2016	Site 14	8.14	6.2	16.0	10.0	10.0	0.0	3.0
22/09/2016	Site 17	7.24	5.3	16.0	10.0	25.0	0.0	6.0
29/09/2016	Site 5	7.95	6.2	16.3	10.0	0.0	0.0	3.0
29/09/2016	Site 7	8.04	7.8	15.7	10.0	10.0	0.0	0.5
29/09/2016	Site 11	8.16	6.7	16.7	10.0	10.0	0.0	0.5
29/09/2016	Site 14	8.21	6.9	16.5	10.0	0.0	0.0	3.0
29/09/2016	Site 17	8.16	7.1	16.4	10.0	0.0	0.0	3.0
06/10/2016	Site 5	7.98	7.4	11.3	10.0	10.0	0.0	3.0
06/10/2016	Site 7	8.16	10.9	10.3	10.0	0.0	0.0	6.0
06/10/2016	Site 11	8.09	6.0	12.9	10.0	0.0	0.0	6.0
06/10/2016	Site 14	8.14	5.2	12.5	10.0	10.0	0.0	0.5
06/10/2016	Site 17	8.21	6.9	12.2	10.0	0.0	0.0	0.5
12/10/2016	Site 5	6.96	6.8	10.2	10.0	10.0	0.0	3.0
12/10/2016	Site 7	7.75	9.7	11.4	10.0	0.0	0.0	3.0
12/10/2016	Site 11	7.84	6.7	11.9	10.0	0.0	0.0	6.0
12/10/2016	Site 14	7.87	6.2	11.5	10.0	0.0	0.0	6.0
12/10/2016	Site 17	7.88	6.5	11.1	10.0	25.0	0.0	6.0
20/10/2016	Site 5	6.98	7.2	10.2	10.0	25.0	0.0	6.0
20/10/2016	Site 7	7.23	9.1	11.2	10.0	10.0	0.0	6.0
20/10/2016	Site 11	8	7.2	10.8	10.0	10.0	0.0	6.0
20/10/2016	Site 14	8.09	7.1	10.7	10.0	10.0	0.0	6.0
20/10/2016	Site 17	8.01	7.1	10.6	10.0	25.0	0.0	6.0
03/11/2016	Site 5	7.92	6.7	8.2	10.0	0.0	0.0	6.0
03/11/2016	Site 7	8.11	11.4	9.6	10.0	10.0	0.0	6.0
03/11/2016	Site 11	8.09	7.1	9.0	10.0	10.0	0.0	6.0
03/11/2016	Site 14	8	6.6	9.2	10.0	0.0	0.0	3.0
03/11/2016	Site 17	7.9	6.3	9.2	10.0	0.0	0.0	3.0
09/11/2016	Site 5	7.79	8.1	9.4	10.0	10.0	0.0	3.0
09/11/2016	Site 7	7.8	8.8	9.4	10.0	0.0	0.0	0.5
09/11/2016	Site 11	8.02	9.2	9.9	10.0	10.0	0.0	0.5
09/11/2016	Site 14	8.06	9.3	9.8	10.0	0.0	0.0	0.5
09/11/2016	Site 17	8.09	9.0	9.0	10.0	0.0	0.0	3.0
17/11/2016	Site 5	7.98	8.1	9.4	10.0	0.0	0.0	3.0
17/11/2016	Site 7	7.95	8.8	9.4	10.0	0.0	0.0	6.0

17/11/2016	Site 11	8.1	9.2	9.9	10.0	0.0	0.0	0.5
17/11/2016	Site 14	8.1	9.3	9.8	10.0	10.0	0.0	3.0
17/11/2016	Site 17	8.05	9.0	9.0	10.0	0.0	0.0	3.0
23/11/2016	Site 5	7.15	9.4	7.7	10.0	10.0	0.0	0.5
23/11/2016	Site 7	7.46	9.3	9.2	10.0	0.0	0.0	0.5
23/11/2016	Site 11	7.57	9.3	7.8	10.0	0.0	0.0	0.5
23/11/2016	Site 14	7.41	9.0	7.4	10.0	0.0	0.0	3.0
23/11/2016	Site 17	7.69	9.1	7.4	10.0	10.0	0.0	3.0
01/12/2016	Site 5	7.89	13.2	2.3	10.0	0.0	0.0	6.0
01/12/2016	Site 7	8.01	10.8	1.2	10.0	0.0	0.0	3.0
01/12/2016	Site 11	7.64	13.1	2.5	10.0	10.0	0.0	0.5
01/12/2016	Site 14	7.78	13.9	2.1	10.0	10.0	0.0	3.0
01/12/2016	Site 17	7.95	14.4	2.0	10.0	0.0	0.0	0.7

na = no analysis.

Table S4. Intracellular microcystin concentrations (μg/L) quantified in water samples from Portishead Lake.

Date		MC-LR	MC-RR	MC-LA	MC-LY	MC-LF	MC-LW	MC-YR	MC-WR	dmMC-LR	MC-HilR	MC-HtyR	D-Asp3-MC-RR	Total MC
21/01/2016	Site 5	21.45	34.45	15.45	0.02	1.23	1.17	3.48	25.09	0.74	0.55	0.03		103.7
	Site 7	73.87	131.98	46.41	0.15	3.65	3.56	11.52	88.92	2.28	1.74	0.07		364.2
	Site 11	3.44	8.99	2.94		0.19	0.20	0.33	4.77	0.12	0.11			21.1
	Site 14	2.11	5.26	1.82		0.12	0.12	0.26	2.79	0.06	0.05			12.6
	Site 17								0.01					0.01
28/01/2016	Site 5	0.02							0.06					0.1
	Site 7													
	Site 11		0.02						0.06					0.1
	Site 14	0.04	0.03	0.02					0.07	0.02				0.2
	Site 17	0.02	0.02						0.06					0.1
03/02/2016	Site 5	0.02	0.01	0.02					0.02					0.1
	Site 7													
	Site 11	0.02	0.01	0.02				0.002	0.02					0.1
	Site 14	0.01	0.01	0.01					0.03					0.1
	Site 17	0.03	0.02	0.01				0.004	0.04					0.1
11/02/2016	Site 5	0.01	0.02											0.03
	Site 7													
	Site 11	0.04	0.04	0.02				0.01	0.03		0.01			0.2
	Site 14	1.41	0.78	1.26		0.06		0.12	1.60	0.06	0.04			5.3
	Site 17	0.01	0.02	0.01										0.04
18/02/2016	Site 5	0.01	0.02	0.01					0.02					0.1
	Site 7													
	Site 11	0.01	0.02	0.01					0.02					0.1
	Site 14	1.73	0.84	1.53		0.09		0.16	2.00	0.06	0.05			6.5
	Site 17	0.01	0.02						0.01					0.04
25/02/2016	Site 5	0.72	0.42	0.43		0.02		0.16	0.46	0.03	0.02			2.3
	Site 7	0.01	0.02	0.01										0.04
	Site 11	0.02	0.02	0.01				0.01	0.02					0.1
	Site 14	0.06	0.05	0.01				0.03	0.02					0.2
	Site 17	7.91	6.23	0.96	0.01	0.05		2.75	1.56	0.32	0.10	0.02		19.9
04/03/2016	Site 5	0.02	0.05	0.01					0.01					0.1
	Site 7	0.01	0.04	0.01										0.1
	Site 11	0.02	0.05	0.01				0.01	0.02					0.1
	Site 14	0.03	0.05	0.03				0.01	0.03					0.2
	Site 17	0.52	0.22	0.33		0.03		0.07	0.36	0.02	0.02			1.6
08/03/2016	Site 5	0.01	0.03			0.01	0.02							0.1
	Site 7					0.01								0.01

	Site 11	0.01	0.03			0.01							0.1
	Site 14	0.02	0.04				0.02	0.01	0.01				0.1
	Site 17		0.03			0.01							0.04
17/03/2016	Site 5	0.54	0.48	0.21				0.15	0.20	0.03	0.01		1.6
	Site 7												
	Site 11	0.01	0.02	0.01									0.04
	Site 14	0.01	0.01						0.01				0.03
	Site 17	0.02	0.02	0.01					0.02				0.1
24/03/2016	Site 5	0.03	0.05	0.01				0.01	0.02				0.1
	Site 7	0.01	0.04										0.1
	Site 11	0.02	0.04	0.01				0.01	0.02				0.1
	Site 14	0.02	0.04	0.01					0.02				0.1
	Site 17	0.03	0.05	0.02				0.01	0.04		0.01		0.2
30/03/2016	Site 5	0.01	0.04	0.01					0.02				0.1
	Site 7												
	Site 11	0.03	0.05	0.02				0.01	0.04	0.01	0.01		0.2
	Site 14	0.06	0.05	0.05				0.02	0.07	0.01			0.3
	Site 17	0.02	0.05	0.03				0.01	0.03				0.1
07/04/2016	Site 5	0.03	0.02	0.01					0.02				0.1
	Site 7	0.03	0.02	0.01					0.01				0.1
	Site 11	0.04	0.03	0.02				0.01	0.04	0.01			0.2
	Site 14	0.02	0.02	0.01				0.01	0.02				0.1
	Site 17	0.11	0.05	0.10				0.02	0.14	0.01			0.4
13/04/2016	Site 5	0.02	0.02	0.01	0.00	0.01		0.01	0.01				0.1
	Site 7	0.01				0.01							0.02
	Site 11	0.01											0.01
	Site 14	0.01	0.02	0.01									0.04
	Site 17	0.40	0.28	0.23			0.01	0.07	0.31	0.03	0.01		1.3
21/04/2016	Site 5	19.15	35.11	6.93		0.49		5.83	12.51	1.15	0.53	0.08	81.8
	Site 7	0.12	0.12	0.04				0.04	0.07	0.00	0.01		0.4
	Site 11	0.02	0.08	0.01				0.01	0.01				0.1
	Site 14	0.02	0.07	0.01				0.01	0.01				0.1
	Site 17	0.02	0.08	0.02				0.01	0.01				0.1
27/04/2016	Site 5	0.02	0.02	0.02		0.01		0.01	0.01				0.1
	Site 7		0.01										0.01
	Site 11		0.01										0.0
	Site 14	10.41	6.46	5.95		0.37	0.37	3.05	10.02	0.94	0.33		37.9
	Site 17	0.04	0.02	0.03				0.01	0.03				0.1
05/05/2016	Site 5	212.0	104.0	121.4		7.8	6.2	64.9	217.0	19.4	7.7	0.7	761.0
	Site 7	0.02	0.01										0.03
	Site 11	0.02	0.01	0.01									0.04
	Site 14	0.02	0.01				0.02	0.00					0.1
	Site 17	0.02	0.02	0.01			0.02	0.00	0.01				0.1

12/05/2016	Site 5	1066.70	581.53	960.71	7.21	44.34	32.35	71.05	1162.65	48.44	35.59	1.70	4012
	Site 7	0.02	0.01	0.03		0.03			0.03				0.1
	Site 11	0.07	0.05	0.05		0.02		0.03	0.04				0.3
	Site 14	0.02	0.01					0.02					0.05
	Site 17	0.08	0.06	0.04				0.03	0.04	0.02			0.3
18/05/2016	Site 5	0.02	0.08	0.02					0.05				0.2
	Site 7												
	Site 11	0.02	0.08	0.01					0.04				0.1
	Site 14	10.46	5.49	8.18		0.58		2.28	7.18	1.00	0.42	0.04	35.6
	Site 17	0.03	0.08	0.04				0.03	0.05				0.2
25/05/2016	Site 5	125.77	92.54	64.97		3.04		51.88	68.69	8.86	3.05	0.31	419
	Site 7	0.02	0.03	0.03				0.02	0.02				0.1
	Site 11	0.02	0.03	0.02				0.02	0.01				0.1
	Site 14		0.03	0.02					0.01				0.1
	Site 17	0.02	0.03	0.02					0.01				0.1
01/06/2016	Site 5	8.57	6.56	8.63		0.78	0.89	1.86	10.27	0.95	0.54	0.29	39.4
	Site 7												
	Site 11												
	Site 14	0.04		0.04									0.1
	Site 17	0.92	0.77	0.94		0.36		0.43	1.13	0.32	0.19		5.1
08/06/2016	Site 5	1.39	1.23	1.00		0.39		0.63	1.09	0.40	0.20		6.3
	Site 7												
	Site 11	0.04	0.06										0.1
	Site 14	0.05	0.06	0.04				0.05	0.08				0.3
	Site 17	1.86	1.69	1.46		0.64		0.79	1.66	0.61	0.30		9.0
16/06/2016	Site 5	47.54	161.89	4.63		0.34	0.22	17.55	6.74	3.86	0.75	0.18	243.7
	Site 7												
	Site 11	0.02	0.05					0.02	0.02				0.1
	Site 14	0.01	0.05	0.01			0.02		0.02				0.1
	Site 17	0.02	0.05	0.01				0.02	0.02				0.1
22/06/2016	Site 5												
	Site 7												
	Site 11	290.45	1183.32	0.66			0.18	110.84	15.50	15.52	3.06	0.18	1619.7
	Site 14	0.03	0.06					0.02	0.02				0.1
	Site 17							0.01					0.01
29/06/2016	Site 5												
	Site 7												
	Site 11	0.03	0.02					0.02	0.02				0.1
	Site 14		0.01										0.01
	Site 17		0.01										0.01
04/07/2016	Site 5												
	Site 7												

	Site 11	0.01	0.01									0.02
	Site 14	0.05	0.05					0.04	0.02			0.2
	Site 17		0.01					0.02				0.03
12/07/2016	Site 5											
	Site 7											
	Site 11	0.02						0.02				0.04
	Site 14	0.10	0.07					0.06	0.02	0.01		0.3
	Site 17	0.02	0.01									0.03
21/07/2016	Site 5	0.01										0.01
	Site 7											
	Site 11	0.01	0.08									0.1
	Site 14		0.08									0.1
	Site 17	0.06	0.13	0.02				0.04				0.2
27/07/2016	Site 5	0.01	0.08					0.01				0.1
	Site 7											
	Site 11	0.02	0.08					0.01				0.1
	Site 14											
	Site 17	0.70	1.08	0.25		0.14		0.34	0.51		0.13	3.1
03/08/2016	Site 5											
	Site 7											
	Site 11											
	Site 14											
	Site 17											
10/08/2016	Site 5											
	Site 7											
	Site 11					0.02						0.02
	Site 14	0.02	0.01									0.03
	Site 17	0.05	0.05					0.02				0.1
18/08/2016	Site 5	0.34	0.24					0.10	0.03	0.01		0.7
	Site 7											
	Site 11	0.02						0.02				0.04
	Site 14	0.01	0.01					0.02				0.04
	Site 17	0.57	0.60					0.44				1.6
25/08/2016	Site 5	67.35	562.27	1.84	0.33			26.40	7.77	3.23	1.28	670.5
	Site 7											
	Site 11	0.36	0.97					0.22	0.14			1.7
	Site 14	0.01						0.01				0.02
	Site 17	1.11	3.23					0.53	0.14			5.0
01/09/2016	Site 5	0.02										0.02
	Site 7											
	Site 11	125.82	808.02	0.21	0.25	0.44	0.29	48.01	8.92	4.97	1.73	999
	Site 14	11.57	41.66				0.21	4.55	0.81	0.95	0.16	59.9
	Site 17	0.15	0.11					0.08	0.02			0.4

08/09/2016	Site 5	0.08	0.12											0.2
	Site 7													
	Site 11	0.02	0.03											0.05
	Site 14	0.95	0.81				0.07	0.28						2.1
	Site 17	1.52	1.82					0.34		0.29				4.0
15/09/2016	Site 5	1259.83	3950.94				0.46	433.77	62.84	20.24				5728
	Site 7													
	Site 11	6.93	11.99					1.90		1.24				22.1
	Site 14	70.08	179.11				0.33	26.89	3.09	1.99				281.5
	Site 17	90.37	106.41	1.14		0.36	0.49	34.20	5.60	3.10				241.7
22/09/2016	Site 5	642.23	937.92	4.47	1.57	5.55	5.24	314.78	53.33	11.91	10.71	1.09	2.51	1991
	Site 7													
	Site 11													
	Site 14													
	Site 17	23.91	42.93					10.79	5.26					82.9
29/09/2016	Site 5													
	Site 7													
	Site 11	0.01	0.02					0.01						0.04
	Site 14	0.02	0.02					0.01						0.1
	Site 17	0.03	0.03					0.01						0.1
06/10/2016	Site 5	1.05	1.21					0.54	0.14	0.19				3.1
	Site 7													
	Site 11	0.05	0.05											0.1
	Site 14	0.01	0.01			0.02								0.04
	Site 17	0.02	0.03					0.02						0.1
12/10/2016	Site 5	8.91	6.32			0.41		3.38	0.84	0.27				20.1
	Site 7													
	Site 11	0.04	0.05					0.02						0.1
	Site 14	0.01	0.01											0.02
	Site 17	0.01	0.01											0.02
20/10/2016	Site 5	21.40	20.80	0.30				8.10	1.70	0.80			0.30	53.4
	Site 7													
	Site 11	0.01	0.00											0.01
	Site 14	0.01	0.00					0.01						0.02
	Site 17	32.30	28.67					10.40	2.30	1.80				75.5
03/11/2016	Site 5													
	Site 7													
	Site 11													
	Site 14													
	Site 17							0.14						0.1
09/11/2016	Site 5													
	Site 7													
	Site 11													

	Site 14
	Site 17
17/11/2016	Site 5
	Site 7
	Site 11
	Site 14
	Site 17
23/11/2016	Site 5
	Site 7
	Site 11
	Site 14
	Site 17
01/12/2016	Site 5
	Site 7
	Site 11
	Site 14
	Site 17

Table S5. Summary of extracellular microcystin concentrations quantified in lake water samples

Date	Site	MC-LR	MC-RR	MC-LA	MC-LY	MC-LF	MC-LW	MC-YR	MC-WR	dmMC-LR	MC-HilR	MC-HtyR	D-Asp3-MC-RR	Total MC
21/01/2016	Site 5	0.7	2.9	0.1					1.1					4.8
	Site 7	0.7	2.9	0.3					0.8					4.7
	Site 11	0.4	1.5	0.2										2.1
	Site 14	0.3	3.3						0.7					4.3
	Site 17													
28/01/2016	Site 5													
	Site 7													
	Site 11													
	Site 14													
	Site 17													
03/02/2016	Site 5													
	Site 7													
	Site 11													
	Site 14													
	Site 17													
11/02/2016	Site 5													
	Site 7													
	Site 11													
	Site 14	0.4	0.85											1.3
	Site 17													
18/02/2016	Site 5													
	Site 7													
	Site 11													
	Site 14	0.41	0.87	0.26										1.5
	Site 17													
25/02/2016	Site 5		0.82											0.8
	Site 7													
	Site 11													
	Site 14													
	Site 17	1.18	1.23	0.6				0.46	1.09					4.6
04/03/2016	Site 5													
	Site 7													
	Site 11													
	Site 14													
	Site 17													
08/03/2016	Site 5													
	Site 7													
	Site 11													
	Site 14													

	Site 17														
17/03/2016	Site 5b		0.53												0.5
	Site 7														
	Site 11														
	Site 14														
	Site 17														
24/03/2016	Site 5														
	Site 7														
	Site 11														
	Site 14														
	Site 17														
30/03/2016	Site 5					0.81									0.8
	Site 7														
	Site 11														
	Site 14														
	Site 17														
07/04/2016	Site 5														
	Site 7														
	Site 11														
	Site 14														
	Site 17														
13/04/2016	Site 5														
	Site 7														
	Site 11														
	Site 14			0.19											0.2
	Site 17			0.15											0.2
21/04/2016	Site 5														
	Site 7														
	Site 11														
	Site 14														
	Site 17														
27/04/2016	Site 5														
	Site 7														
	Site 11														
	Site 14														
	Site 17														
05/05/2016	Site 5	2.61	1.43	1.64				0.48	2.53						8.7
	Site 7														
	Site 11														
	Site 14														
	Site 17														
12/05/2016	Site 5	0.90	0.24	4.53				0.65	0.80						7.1
	Site 7						0.71								0.7

	Site 11	0.57												0.6
	Site 14													
	Site 17	0.55												0.6
18/05/2016	Site 5													0.0
	Site 7													0.0
	Site 11													
	Site 14	2.09	3.86	2.01				1.2	2.75					11.9
	Site 17													
25/05/2016	Site 5	6.35	3.32	3.73				1.73	2.17		0.67			18.0
	Site 7													
	Site 11													
	Site 14													
	Site 17													
01/06/2016	Site 5	1.68		1.85										3.5
	Site 7													
	Site 11													
	Site 14													
	Site 17													
08/06/2016	Site 5			1.68										1.7
	Site 7													
	Site 11													
	Site 14													
	Site 17													
16/06/2016	Site 5	0.66	2.23					0.62		0.97				4.5
	Site 7			0.51				0.59						1.1
	Site 11													
	Site 14						0.83							0.8
	Site 17													
22/06/2016	Site 5													
	Site 7													
	Site 11	8.72	8.05					2.41	0.73	1.63				21.5
	Site 14	0.63	2.18					0.64						3.4
	Site 17													
29/06/2016	Site 5													
	Site 7													
	Site 11													
	Site 14													
	Site 17													
04/07/2016	Site 5													
	Site 7													
	Site 11													
	Site 14													

	Site 17			
12/07/2016	Site 5			
	Site 7			
	Site 11			
	Site 14			
	Site 17			
21/07/2016	Site 5			
	Site 7			
	Site 11			
	Site 14			
	Site 17			
27/07/2016	Site 5			
	Site 7			
	Site 11			
	Site 14			
	Site 17	0.73	0.75	1.47
03/08/2016	Site 5			
	Site 7			
	Site 11			
	Site 14			
	Site 17			
10/08/2016	Site 5			
	Site 7			
	Site 11			
	Site 14			
	Site 17			
18/08/2016	Site 5			
	Site 7			
	Site 11			
	Site 14			
	Site 17			
25/08/2016	Site 5		0.17	0.17
	Site 7			
	Site 11			
	Site 14			
	Site 17			
01/09/2016	Site 5			
	Site 7			
	Site 11	0.49		0.49
	Site 14			
	Site 17			
08/09/2016	Site 5			
	Site 7			

	Site 11			
	Site 14			
	Site 17			
15/09/2016	Site 5	1.12	2.07	3.19
	Site 7			
	Site 11			
	Site 14			
	Site 17			
22/09/2016	Site 5		1.19	1.19
	Site 7			
	Site 11			
	Site 14			
	Site 17			
29/09/2016	Site 5			
	Site 7			
	Site 11			
	Site 14			
	Site 17			
06/10/2016	Site 5			
	Site 7			
	Site 11			
	Site 14			
	Site 17			
12/10/2016	Site 5			
	Site 7			
	Site 11			
	Site 14			
	Site 17			
20/10/2016	Site 5			
	Site 7			
	Site 11			
	Site 14			
	Site 17			
03/11/2016	Site 5			
	Site 7			
	Site 11			
	Site 14			
	Site 17			
09/11/2016	Site 5			
	Site 7			
	Site 11			
	Site 14			
	Site 17			

17/11/2016	Site 5
	Site 7
	Site 11
	Site 14
	Site 17
23/11/2016	Site 5
	Site 7
	Site 11
	Site 14
	Site 17
01/12/2016	Site 5
	Site 7
	Site 11
	Site 14
	Site 17

Table S6. Parsimonious linear mixed-effect model output.

```
Linear mixed model fit by REML. t-tests use Satterthwaite's method ['lmer-
ModLmerTest']
Formula: log_MC_Int ~ Nitrate + Phosphate + pH + DO + Total rain + Av wind speed +
(1 | Site)
   Data: environmental_factors

REML criterion at convergence: 217.7

Scaled residuals:
    Min      1Q  Median      3Q     Max
-1.3787 -0.7975 -0.1093  0.5781  2.5062

Random effects:
 Groups   Name        Variance Std.Dev.
 Site     (Intercept) 0.3134   0.5598
 Residual             0.7957   0.8920
Number of obs: 71, groups:  Site, 6

Fixed effects:
             Estimate Std. Error        df t value Pr(>|t|)
(Intercept) 14.409876   2.943814 63.943255   4.895 6.99e-06 ***
Nitrate      0.011911   0.004861 61.955173   2.450   0.0171 *
Phosphate    0.018444   0.014715 59.411378   1.253   0.2150
pH          -1.881192   0.357652 63.844870  -5.260 1.79e-06 ***
DO          0.024836   0.066890 62.647186   0.371   0.7117
Total rain   0.005116   0.010538 58.354796   0.485   0.6292
Av wind speed    -0.060970   0.135755 58.788792  -0.449   0.6550
---
Signif. codes:  0 ‘***’ 0.001 ‘**’ 0.01 ‘*’ 0.05 ‘.’ 0.1 ‘ ’ 1

Correlation of Fixed Effects:
           (Intr) Nitrat Phspht pH     DO    Ttl_rn
Nitrate    -0.631
Phosphate  -0.103  0.150
pH         -0.961  0.515  0.057
DO        -0.172  0.362 -0.126 -0.044
Total rain -0.097  0.050  0.045  0.021  0.297
Av wind speed   -0.094  0.183  0.093 -0.031 -0.014 -0.209
```

Figure S2. Parsimonious linear mixed-effect model residual plot.

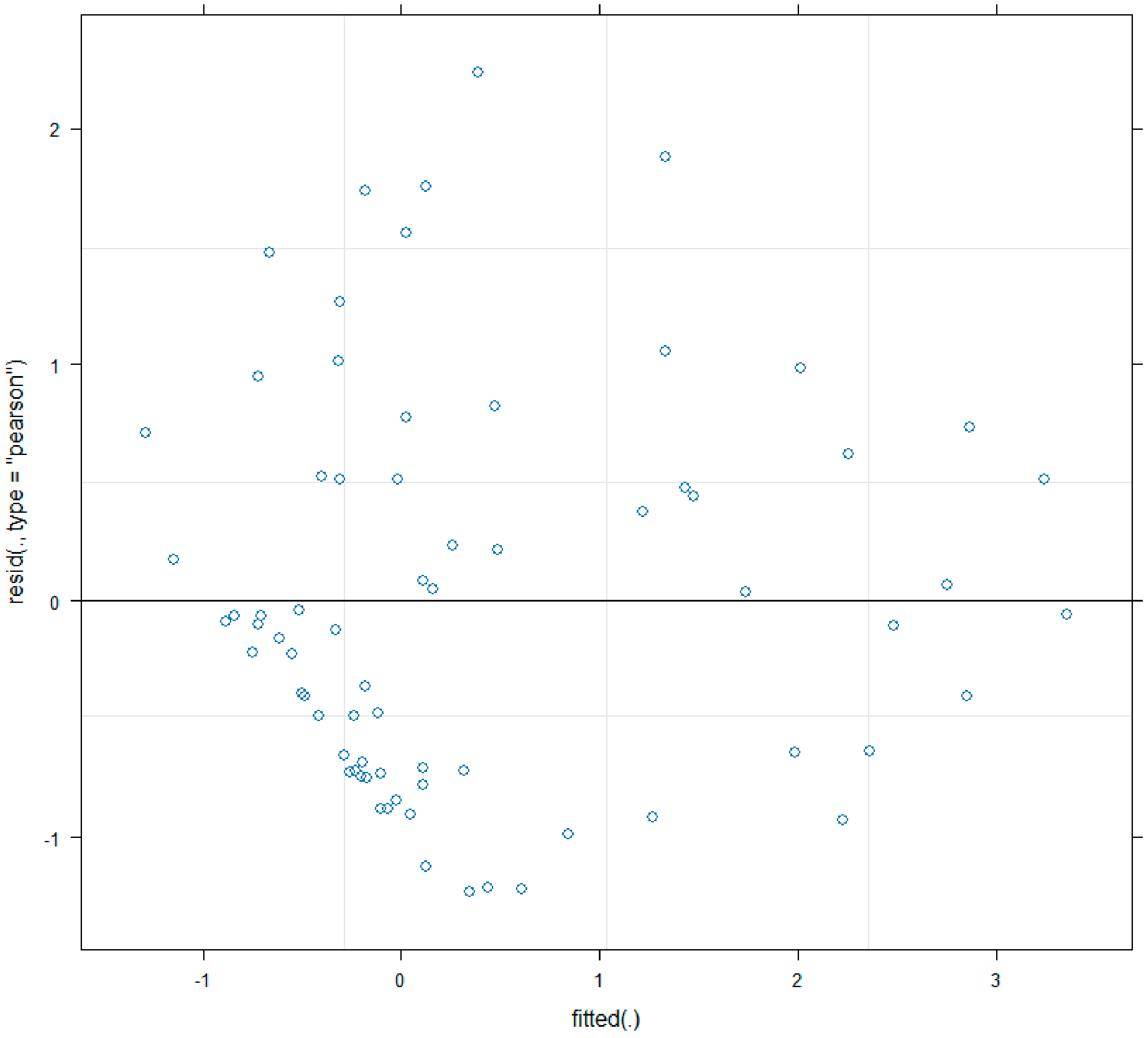

Figure S3. Site effect on log intracellular MC concentrations from linear mixed-effect model output.

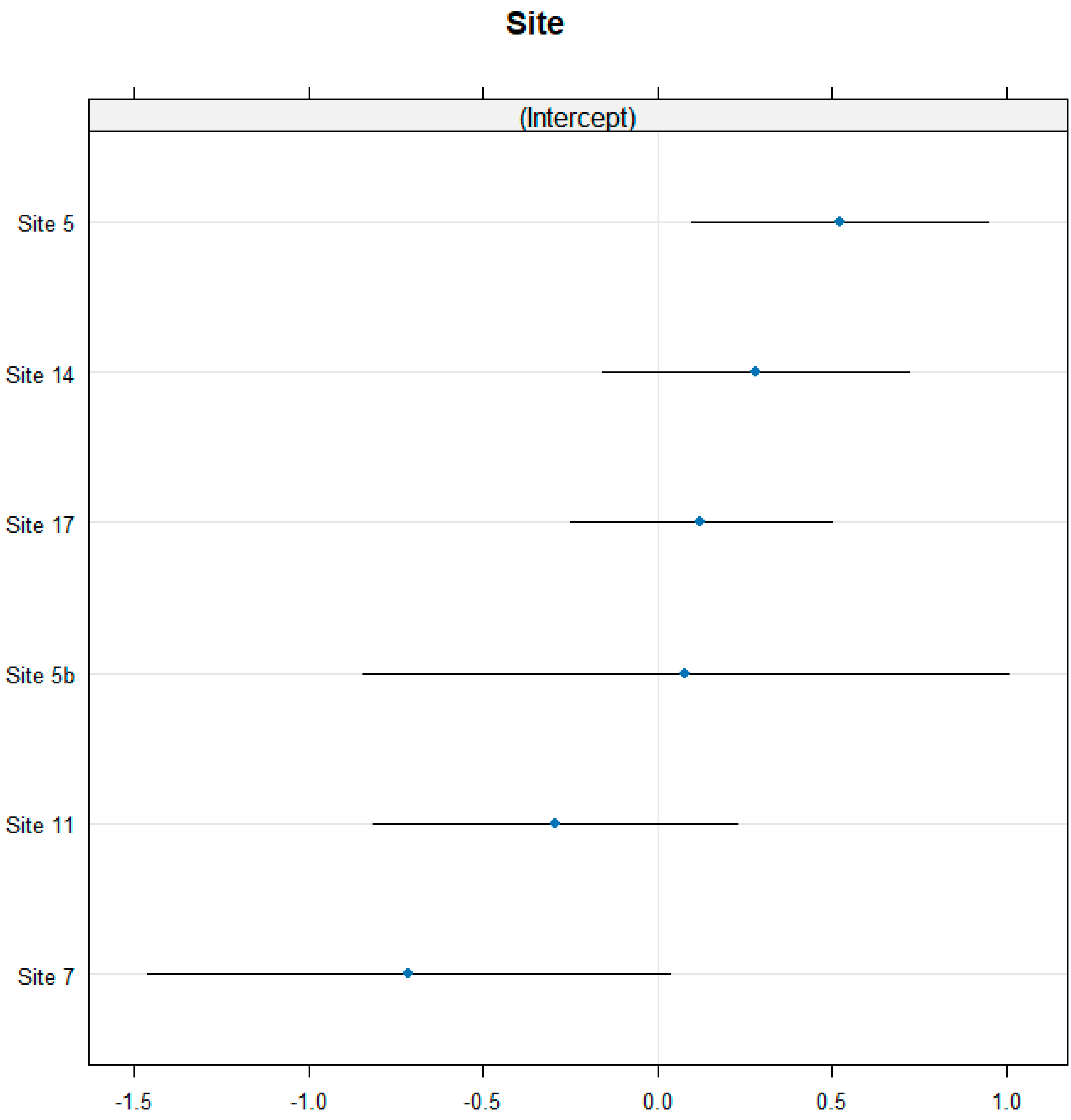

Figure S4. Random Forest variable importance plot.

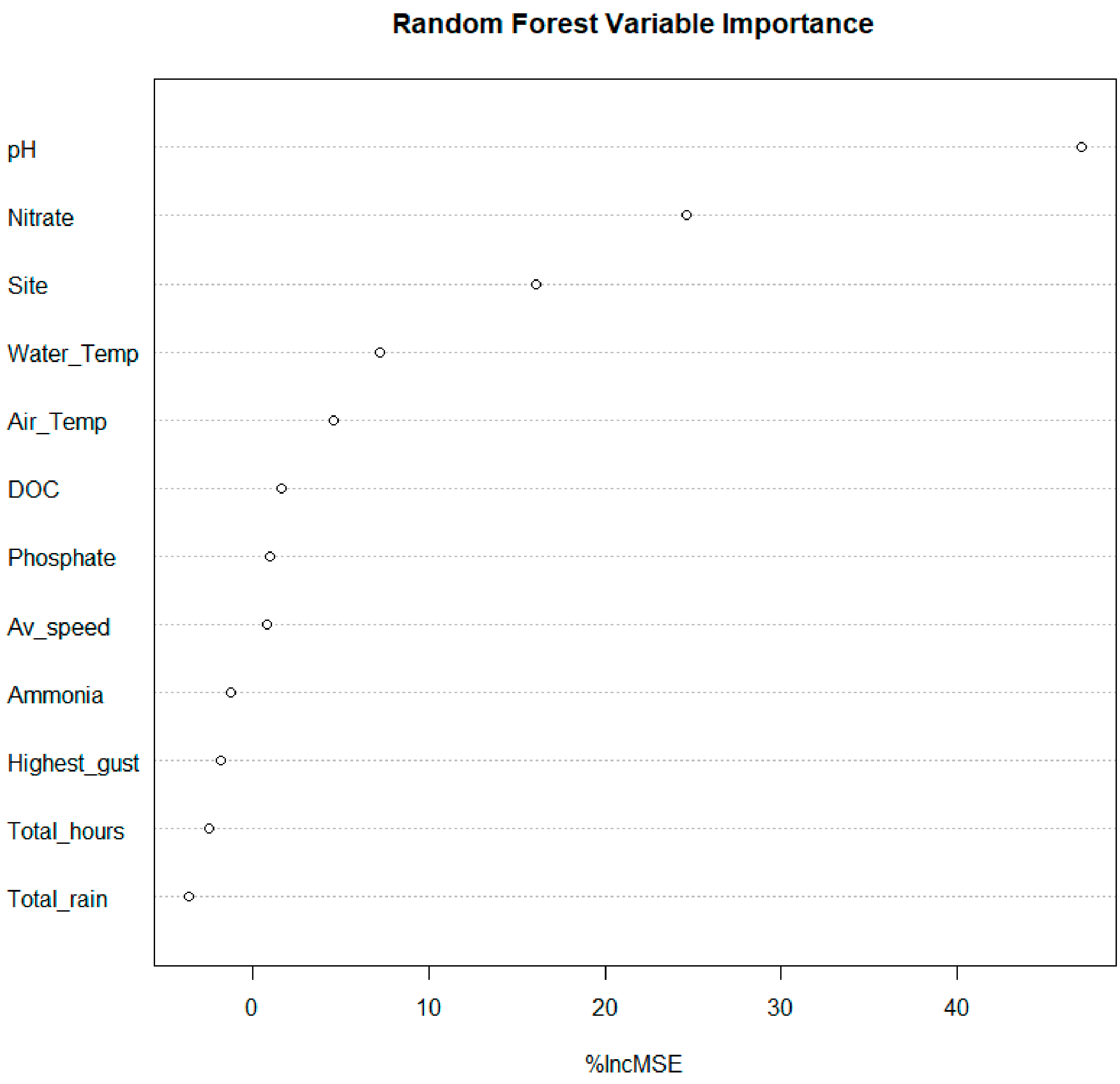

Figure S5. Parsimonious linear model output and ANOVA on effect of site on MC concentrations.

Anova-

```
 Response: log_MC_Int
           Df Sum Sq Mean Sq F value    Pr(>F)
Site        5 35.765   7.153  9.0903 1.886e-06 ***
Nitrate     1 38.612  38.612 49.0695 2.654e-09 ***
Phosphate   1  2.187   2.187  2.7792    0.1008
pH          1 20.172  20.172 25.6350 4.329e-06 ***
DO          1  0.359   0.359  0.4564    0.5019
Total_rain  1  0.143   0.143  0.1818    0.6714
Av_speed    1  0.036   0.036  0.0452    0.8323
Residuals  59 46.426   0.787
---
Signif. codes:  0 ‘***’ 0.001 ‘**’ 0.01 ‘*’ 0.05 ‘.’ 0.1 ‘
’ 1
```

Linear model -

```
lm(formula = log_MC_Int ~ Site + Nitrate + Phosphate + pH + DO +
    Total_rain + Av_speed, data = environmental_factors)


Residuals:
     Min       1Q   Median       3Q      Max
-1.33886 -0.63018 -0.06105  0.47132  2.11666


Coefficients:
             Estimate Std. Error t value Pr(>|t|)
(Intercept) 13.657503   3.043341   4.488 3.39e-05 ***
SiteSite 14  0.524015   0.350301   1.496  0.14001
SiteSite 17  0.454084   0.342375   1.326  0.18986
SiteSite 5   0.784612   0.369744   2.122  0.03804 *
SiteSite 5b  0.259023   0.945219   0.274  0.78502
SiteSite 7  -1.399262   0.606985  -2.305  0.02469 *
Nitrate      0.013777   0.004935   2.791  0.00706 **
Phosphate    0.019932   0.014749   1.351  0.18172
pH          -1.859402   0.366527  -5.073 4.17e-06 ***
DO           0.052473   0.068081   0.771  0.44394
Total_rain   0.004858   0.010521   0.462  0.64597
Av_speed    -0.028911   0.135946  -0.213  0.83232
---
Signif. codes:  0 ‘***’ 0.001 ‘**’ 0.01 ‘*’ 0.05 ‘.’ 0.1 ‘ ’ 1
```

```
Residual standard error: 0.8871 on 59 degrees of freedom
  (14 observations deleted due to missingness)
Multiple R-squared:  0.6769,    Adjusted R-squared:  0.6167
F-statistic: 11.24 on 11 and 59 DF,  p-value: 7.263e-11
```

Figure S6. Post hoc Tukey's analysis of site effect on MC concentrations.

contrast	estimate	SE	df	t.ratio	p.value
Site 11 - Site 14	-0.5240	0.350	59	-1.496	0.6682
Site 11 - Site 17	-0.4541	0.342	59	-1.326	0.7694
Site 11 - Site 5	-0.7846	0.370	59	-2.122	0.2904
Site 11 - Site 5b	-0.2590	0.945	59	-0.274	0.9998
Site 11 - Site 7	1.3993	0.607	59	2.305	0.2084
Site 14 - Site 17	0.0699	0.315	59	0.222	0.9999
Site 14 - Site 5	-0.2606	0.374	59	-0.696	0.9817
Site 14 - Site 5b	0.2650	0.937	59	0.283	0.9997
Site 14 - Site 7	1.9233	0.596	59	3.228	0.0238*
Site 17 - Site 5	-0.3305	0.326	59	-1.014	0.9116
Site 17 - Site 5b	0.1951	0.926	59	0.211	0.9999
Site 17 - Site 7	1.8533	0.599	59	3.096	0.0339*
Site 5 - Site 5b	0.5256	0.930	59	0.565	0.9929
Site 5 - Site 7	2.1839	0.596	59	3.663	0.0068*
Site 5b - Site 7	1.6583	1.040	59	1.587	0.6102
